# A Review of Antioxidant Activity, Anti‐Inflammatory Properties, Apoptosis‐Regulatory Effects, and Immune System Modulation of *Juglans regia* L. (Walnut)

**DOI:** 10.1002/fsn3.71081

**Published:** 2025-10-31

**Authors:** Mahboobeh Ghasemzadeh Rahbardar, Mostafa Rashki, Mohammad Hossein Boskabady

**Affiliations:** ^1^ Department of Emergency Medicine Faculty of Medicine, Mashhad University of Medical Sciences Mashhad Iran; ^2^ Clinical Research Development Unit, Shahid Hasheminejad Hospital Mashhad University of Medical Sciences Mashhad Iran; ^3^ Applied Biomedical Research Center, Mashhad University of Medical Sciences Mashhad Iran; ^4^ Department of Physiology Faculty of Medicine, Mashhad University of Medical Sciences Mashhad Iran

**Keywords:** apoptosis, cytokines, free radicals, immunity, inflammation mediators, *Juglans regia* L.

## Abstract

The search for natural sources of antioxidants, anti‐inflammatory, apoptosis‐regulatory compounds, and immunomodulators has gained attention in recent years due to their potential therapeutic benefits in a variety of disorders. *Juglans regia* L. ( *J. regia* L.) or walnut has been identified as a promising candidate, with numerous studies indicating its advantageous effects on human health. This review aims to provide a comprehensive overview of the antioxidant, anti‐inflammatory, apoptosis‐regulatory, and immunomodulatory properties of *J. regia* L. An extensive literature review was conducted using electronic databases to find relevant research on the effects of *J. regia* L. on oxidative stress, inflammation, apoptosis, and immune modulation. The search encompassed articles published up to August 2024, and in vitro, in vivo, and clinical trials were included. Walnut possesses potent antioxidant activity, attributed to its rich content of polyphenols, flavonoids, and other bioactive compounds. These components have been demonstrated to scavenge free radicals, alleviate oxidative stress, and prevent cellular damage. *J. regia* L. inhibits pro‐inflammatory mediators and pathways, including cytokines, cyclooxygenase enzymes, and NF‐κB activation. It also exhibits apoptosis‐regulatory properties, potentially influencing programmed cell death pathways. Furthermore, walnut has immunomodulatory properties that regulate immune cell function, modulate cytokine production, and enhance immunological responses. *J. regia* L. represents a valuable natural source of antioxidants, anti‐inflammatory agents, apoptosis‐regulatory agents, and immunomodulators. The walnut's rich phytochemical profile contributes to its therapeutic potential in combating oxidative stress and inflammation, regulating apoptosis and immune responses. More research is needed to understand the underlying mechanisms and maximize the use of walnut‐based therapies for the prevention and treatment of a variety of disorders linked to oxidative damage, inflammation, apoptosis, and immunological dysregulation.

## Graphical abstract

*J. regia* L. represents a valuable natural source of antioxidants, anti‐inflammatory agents, apoptosis‐regulatory agents, and immunomodulators. The walnut‐rich phytochemical profile contributes to its therapeutic potential in combating oxidative stress and inflammation, regulating apoptosis and immune responses.

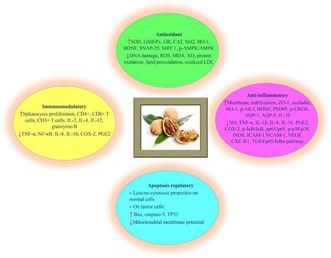

**Abbreviations**
5‐HT: 5‐hydroxytryptamine
Ach: acetylcholine
AChE: acetylcholinesterase
ALP: alkaline phosphatase
ALT: alanine transaminase
AOPP: advanced oxidation protein product
AQP: aquaporin
AST: aspartate aminotransferase
ATG7: autophagy‐related gene 7
ATP: adenosine triphosphate
Aβ: amyloid beta
BALF: broncho‐alveolar lavage fluid
Bax: Bcl‐2‐associated X protein
Bcl‐2: B‐cell lymphoma 2
BDNF: brain‐derived neurotrophic factor
BV‐2: bone marrow‐derived microglial cell line 2
CAT: catalase
CD: cluster of differentiation
CDKN1A: cyclin‐dependent kinase inhibitor 1A
ChAT: choline acetyltransferase
ChREBP: carbohydrate‐responsive element‐binding protein
COX‐2: cyclooxygenase‐2
CREB: cAMP response element‐binding protein
CRP: C‐reactive protein
CXC‐R1: C‐X‐C motif chemokine receptor 1
DNA: deoxyribonucleic acid
ERK: extracellular signal‐regulated kinases
FAS: fatty acid synthase
FBG: fasting blood glucose
FOXO3a: transcription factor forkhead box O3 protein
FRAP: ferric reducing antioxidant potential
G6PC: glucose‐6‐phosphatase
GABA: gamma‐aminobutyric acid
GGT: gamma‐glutamyl transferase
GR: glutathione reductase
GSH: glutathione
GSH‐Px1: glutathione peroxidase 1
GST: glutathione S‐transferase
GSTP: glutathione S‐transferase p
GSTT2: glutathione S‐transferase theta 2
HDL: high‐density lipoprotein
HO‐1: heme oxygenase‐1
HT‐22: hippocampal neuronal cell line 22
ICAM: intracellular adhesion molecule
Ig: immunoglobulin
IL: interleukin
iNOS: inducible nitric oxide synthase
IĸB: NF‐kappa‐B inhibitor
IκBα: inhibitor of nuclear factor kappa B
JAK: Janus kinase
LDH: lactate dehydrogenase
LDL: low‐density lipoprotein
LNCaP: lymph node carcinoma of the prostate
LPS: lipopolysaccharide
MAP1BLC3: microtubule‐associated proteins 1A/1B light chain 3A
MAPKs: p38 mitogen activated protein kinases
MCF‐7: Michigan Cancer Foundation‐7
MCP‐1: monocyte chemoattractant protein‐1
MDA: malondialdehyde
MMP: mitochondrial membrane potential
MnSOD: manganese superoxide dismutase
mRNA: messenger ribonucleic acid
NF‐κB: nuclear factor kappa‐light‐chain‐enhancer of activated B cells
NLRC3: NOD‐like receptor family CARD domain containing 3
NO: nitric oxide
Nrf2: nuclear factor erythroid 2‐related factor 2
p65: nuclear factor kappa B subunit 65
p‐AKT: phosphorylated protein kinase B
p‐AMPK: phosphorylated 5' adenosine monophosphate‐activated protein kinase
PC12: pheochromocytoma 12
PDK1: 3‐phosphoinositide‐dependent kinase 1
PEPCK: phosphoenolpyruvate carboxyl kinase
PGE2: prostaglandin E2
PI3K: phosphoinositide 3‐kinase
p‐JNK: phosphorylated c‐Jun N‐terminal kinase
PKA: protein kinase A
p‐mTOR: phosphorylated mammalian target of rapamycin
p‐p38: phosphorylated p38 mitogen‐activated protein kinase
PSD95: postsynaptic density protein
PTEN: phosphatase and tensin homolog
Ref: references
RHI: reactive hyperemia index
RORC: RAR‐related orphan receptor C
ROS: reactive oxygen species
SIRT1: sirtuin‐1
SNAP‐25: synaptosomal‐associated protein of 25
SOD: superoxide dismutase
STAT: signal transducers and activators of transcription
TBARS: thiobarbituric acid reactive substances
TC: total cholesterol
TG: triglyceride
TGF‐β: transforming growth factor beta
TLR4: toll‐like receptor 4
TNFR1: tumor necrosis factor receptor 1
TNF‐α: tumor necrosis factor alpha
TP53: tumor protein p53
UVB: ultraviolet B
VCAM: vascular cell adhesion molecule
VEGF: vascular endothelial growth factor
VLDL: very‐low‐density lipoprotein
WEE: walnut ethanolic extract
WME: walnut methanolic extract
WPH: walnut protein hydrolysate
XO: xanthine oxidase
ZO‐1: zonula occludens‐1

## 1 Introduction

Oxidative stress, chronic inflammation, and immunological dysregulation are known to play important roles in the development of a variety of diseases, including nervous system disorders (Jalali and Ghasemzadeh 2023), cardiovascular diseases (Ghasemzadeh Rahbardar et al. 2024; Rashki et al. 2025), respiratory system disorders (Ghasemzadeh Rahbardar and Hosseinzadeh 2023; Ghasemzadeh Rahbardar et al. 2023), renal system diseases (Aghadavod et al. 2016; Ebert et al. 2021), cancer (Bardelčíková et al. 2023), pain (Ghasemzadeh Rahbardar and Hosseinzadeh 2024b; Nakisa and Ghasemzadeh 2022), metabolic syndrome (Naraki et al. 2023; Oskouei et al. 2023), cytotoxicity (Xiong et al. 2022), and insomnia (Ghasemzadeh Rahbardar and Hosseinzadeh 2024). In recent decades, the importance of these factors in disease development has received a lot of attention, encouraging researchers to investigate new therapeutic approaches. Herbal medicine, with a wide variety of bioactive chemicals, has emerged as a promising area of medical research, presenting potential treatments for oxidative stress (Ghasemzadeh Rahbardar, Khosravi, et al. 2025; Ghasemzadeh Rahbardar and Hosseinzadeh 2024a), inflammation (Ghasemzadeh Rahbardar, Taghavizadeh Yazdi, et al. 2025), and immunological dysregulation (Alanazi et al. 2023).

Among the herbal medicines gaining popularity, walnut ( *Juglans regia* L.) [the plant name has been checked with http://www.theplantlist.org (List, n.d.)], a deciduous tree of the Juglandaceae family (Chudhary et al. 2020), stands out as a promising alternative. Walnut is native to various regions such as Eastern Asia, Northern America, as well as Southeastern Europe (Pollegioni et al. 2017; Aradhya et al. 2017) and has been used in folk medicine for ages in many nations (Taha and Al‐wadaan 2011; Ebrahimi et al. 2018) with documented applications ranging from curing digestive issues and skin conditions to increasing overall quality of life (Hussain et al. 2021; Croitoru et al. 2019). In traditional Chinese medicine theory, the following properties of walnut green husk have been stated: astringency, bitterness, calmness, pungency, removal of heat and detoxification, wind expulsion, and treatment of ringworm, pain relief, and cessation of dysentery (Li et al. 2024; Wu 1988). Also, walnut leaves are utilized for a range of illnesses due to their extensive applications in traditional medicine. These include hypoglycemic, carminative, astringent, keratolytic, antidiarrheal, antihelmintic, depurative, and tonic. Additionally, they have been used to treat sinusitis, colds, and stomachaches (Mouhajir et al. 2001; Taha and Al‐wadaan 2011; Jaiswal and Tailang 2017). In Turkish ancient medicine, fresh leaves are topically applied to the body or forehead to reduce temperature or to swollen joints to treat rheumatic pain (Yeşilada 2002). Furthermore, the *J. regia* L. kernel has long been used in Iranian medicine for the management of inflammatory bowel disease (Rahimi et al. 2010). Avicenna claimed that several walnut tree parts, including the bark, fruit, and leaves, are useful in treating illnesses like diarrhea, skin disorders, and respiratory problems (Avicenna 2010). Furthermore, Mohammad Hossein Aghili Khorasani highlighted in Makhzan al‐Adwiya the benefits of walnut for health issues related to the digestive system, skin, wound healing, and diabetes (Khorasani, n.d.). Another old book called “Tohfat al‐Momenin” listed the medicinal benefits of walnut for ailments like diarrhea, constipation, and stomach problems. Additionally, it addresses the use of walnut oil to treat skin conditions (Momen Tonekaboni 2010). In Palestine, the leaves are used to cure asthma and diabetes (Kaileh et al. 2007). This medicinal plant is applied topically to cure a number of conditions, such as skin irritation, scrofula, excessive perspiration on the hands and feet, and chronic eczema. Applying its leaves topically helps cure minor burns, itchy scalp, dandruff, and sunburn. They also serve as an additional emollient while treating certain skin conditions. This plant fruit adds to the heart‐protective and bone‐prevention benefits of a diet high in walnuts with its efficient osteoblastic activity and considerable anti‐atherogenic potential. Chinese medicine has historically used the exocarp from immature green fruit, branches, and bark of this medicinal plant to treat stomach, liver, and lung malignancies. Traditional healers in northern Mexico utilize it as liver protection. The bark is made into miswaks, which are used to clean teeth. In Nepal, bark paste is used to cure a variety of skin issues, including toothaches, arthritis, and hair growth. Additionally, the seed coat promotes wound healing. Finally, Calabrian traditional medicine uses the *J. regia* L. shell to treat malaria (Taha and Al‐wadaan 2011; Jaiswal and Tailang 2017). Its historical use in traditional treatments has drawn scientific interest, motivating more research into its pharmacological characteristics and medicinal potential. In addition to its culinary value, walnut has a diverse range of bioactive elements such as polyphenols, flavonoids, phenolic acids, and other phytochemicals (Kafkas et al. 2020; Giura et al. 2019).

Walnuts have a variety of pharmacological effects, particularly antioxidant (Jahanban‐Esfahlan et al. 2019; Mateș et al. 2024), anti‐inflammatory, and immunomodulatory properties (Mobashar et al. 2022). These characteristics have attracted the interest of researchers searching for alternatives for managing oxidative stress, inflammation, and immunological dysregulation diseases. The antioxidant property of walnut stems from its high concentration of polyphenols and flavonoids, which help to scavenge free radicals, reduce oxidative damage, and maintain cellular integrity (Croitoru et al. 2019; Vieira et al. 2019).

Walnut also shows promising anti‐inflammatory properties by regulating several inflammatory mediators and signaling pathways (Fizeșan et al. 2021). It has been demonstrated to suppress the production and release of pro‐inflammatory cytokines, enzymes, and transcription factors, hence reducing the inflammatory response (Laubertová et al. 2015). The anti‐inflammatory properties of walnut make it suitable for the prevention and treatment of disorders marked by excessive or chronic inflammation.

Walnut also has immunoregulatory effects by altering immune cell activity, controlling cytokine production, and increasing immunological responses (Mao et al. 2020). These immunomodulatory properties highlight the potential of walnut as a useful resource for immune‐related disorders and conditions.

This review aims to provide a comprehensive analysis of the current scientific literature concerning the antioxidant, anti‐inflammatory, and immunomodulatory effects of walnut. By exploring the underlying mechanisms through which walnut exerts its beneficial effects, we seek to shed light on its potential as a natural therapeutic agent in modern medicine and pharmacology. Understanding and using the pharmacological properties of walnut may contribute to the development of novel preventive and therapeutic strategies for diseases associated with oxidative stress, inflammation, and immune dysregulation.

## 2 Methods

The literature search was carried out to the end of August 2024, using multiple databases such as PubMed, Scopus, and Google Scholar. The search method included using specific keywords such as “walnut” or “ *Juglans regia* L.” as well as terms like “anti‐inflammatory,” “inflammatory,” “anti‐inflammation,” “oxidative stress,” “oxidative damage,” “apoptosis,” “apoptotic,” “anti‐apoptotic,” and “immunomodulatory,” in the article titles. The search was limited to articles published in peer‐reviewed journals and written in English. The selected studies have the following inclusion criteria: (1) studies focusing on the anti‐inflammatory, antioxidant, or immunomodulatory effects of walnut or its components in in vitro, in vivo, or clinical trial settings; (2) studies that provided information about the underlying mechanisms or pathways associated with these effects; and (3) studies that used pure walnut components or standardized *Juglans regia* L. extracts. Studies that met the exclusion criteria included: (1) studies involving other walnut species; (2) studies combining walnut with other medications or natural compounds; and (3) reviews, meta‐analyses, letters, editorials, or conference abstracts.

## 3 Results

### 3.1 Antioxidant Effects of *Juglans regia* L. and Its Main Components

Oxidative stress develops when reactive oxygen species (ROS) production exceeds the capacity of the antioxidant defense system, causing cellular dysfunction and tissue damage (Afzal et al. 2023). The accumulation of oxidative markers promotes the oxidation of nucleic acids, proteins, and lipids, which contributes to oxidative stress‐related disorders (Dash et al. 2025). Numerous disorders, such as cardiovascular disease (Izzo et al. 2021), diabetes (Kang and Yang 2020), neurodegenerative diseases (Fazeli Kakhki et al. 2024; Yazdanpanah et al. 2023), cancer (Hayes et al. 2020), and aging (Wu et al. 2021), can be triggered by oxidative stress. Natural compounds with antioxidant functions have received attention as likely treatment alternatives for oxidative stress‐related diseases (Rajabalizadeh et al. 2024).

Antioxidants are substances that may mitigate or slow the oxidative damage induced by ROS and other free radicals (Chaudhary et al. 2023). Their role in improving health and preventing disease is critical. Numerous research studies have investigated the antioxidant properties of walnut and its main components.

#### 3.1.1 In Vitro

##### 3.1.1.1 Neurons

It has been reported that pretreatment of Pheochromocytoma 12 (PC12) cells with *J. regia* L. extract before exposure to Amyloid beta‐protein (Aβ) reduced Aβ‐mediated cell death, deoxyribonucleic acid (DNA) damage, lactate dehydrogenase (LDH) release, and ROS generation (Muthaiyah et al. 2011). Pretreating rat primary hippocampal neurons with walnut methanolic extract or polyunsaturated fatty acids (alpha‐linolenic acid, eicosapentaenoic acid, docosahexaenoic acid, and linoleic acid) before exposing them to lipopolysaccharide (LPS) or dopamine revealed that walnut methanolic extract, docosahexaenoic acid, and alpha‐linolenic acid reduced cell death as well as calcium dysregulation. The observed effects were stressor‐dependent and concentration‐dependent (Carey et al. 2013) (Table 1).

**TABLE 1:** The antioxidant effects of *J. regia* L. on in vitro, in vivo, and clinical trials.

Compound	Study design	Doses/duration	Results	Ref.
In vitro				
Walnut extract	PC12 cells	0–4 μg	↓Aβ‐mediated cell death, DNA damage, LDH release, ROS generation	(Muthaiyah et al. 2011)
WME, polyunsaturated fatty acids	Rat primary hippocampal neurons	WME: 0.1, 0.2, 0.5, 1 mg/mL, 30 min	↓Cell death, calcium dysregulation	(Carey et al. 2013)
Walnut peptides (GGW, VYY, LLPF)	PC12 cells	0.10 mM	↑ Cell viability, radical scavenging activity, SOD, GSH‐Px ↓ ROS amounts, apoptosis	(Wang et al. 2018)
WEKPPVSH	BV‐2 microglia	12.5, 25, 50, 100, or 200 μM, 24 h	↑ SOD and CAT activity, expression of Nrf2, HO‐1 ↓ ROS production	(Gao et al. 2021)
TW‐7	HT‐22 Cells	100 μM	↑ Activity of SOD, GSH‐Px, CAT, PINK1‐mediated mitophagy, synaptic function, BDNF and SNAP‐25 levels ↓ Opening of mitochondrial permeability transition pore, mitochondrial bioenergetic deficits, cellular and mitochondrial ROS levels, JNK phosphorylation, mitochondrial apoptosis	(Yang et al. 2022)
SGGY	SH‐SY5Y cells	0.1, 0.5, and 1 mg/mL, 4, 8, 12 h	↑ Cell viability, nuclear translocation of Nrf2 ↓ Oxidative stress, ROS and MDA level, loss of MMP, apoptosis, activation of JNK and MAPKs	(Feng et al. 2023)
Walnut polyphenols and urolithin A	SH‐SY5Y cells	Walnut polyphenols: 50, 100 μg/mL Urolithin A: 5, 10 μM	↑ Cell viability, leakage of extracellular LDH, activity of SOD CAT, cAMP‐dependent PKA, expression of p‐CREB and BDNF ↓ Apoptosis, intracellular calcium, ROS production	(An et al. 2023)
Walnut oil	SH‐SY5Y cells	15.56 μg/mL	↑ ACh level ↓ Oxidative stress, protein oxidation, tau protein levels, AChE activity	(Demirel et al. 2024)
Walnut oil	U937	20–319 μg/mL, 24 h	↑ Antioxidant, SOD activity	(Laubertová et al. 2015)
WEE	Human keratinocytes cell line		↑ GSH ↓ ROS production, lipid peroxidation, inflammatory process	(Calcabrini et al. 2017)
In vivo				
WPH	Rats	666 mg/kg, 25 days, gavage	↑ Behavioral performance, amounts of SOD, GSH‐Px, CAT ↓ MDA	(Wang et al. 2018)
Walnut	AD‐tg mice	9% of diet, 5, 10, 15 months, p.o.	↑ Activity of antioxidant enzymes ↓ ROS level, protein oxidation, lipid peroxidation	(Pandareesh et al. 2018)
Walnut suspension	Albino Wistar rats	400 mg/kg, 4 weeks, p.o.	↑ Memory function, SOD, CAT, GSH‐Px ↓ MDA	(Haider et al. 2018)
Walnut peptides	Mice	666 mg/kg, 21 days, gavage	↑ SOD and CAT activity ↓ Memory deficits, brain oxidative stress and inflammatory response, MDA amounts	(Wang et al. 2020)
Walnut kernel and WSE	Female Wistar rats	9% of the daily diet, 8 weeks, p.o.	↑ Cellular antioxidant activity, GSH ↓ROS, advanced glycation end products, NO, MDA, AChE activity in the brain	(Rusu et al. 2020)
WPH	SPF Kunming mice	333, 666 mg/kg, 21 days, gavage	↑ Behavioral performance, expression of Nrf2, BDNF, CREB, levels of ACh, ACh receptor ↓ AChE activity	(Wang, Su, et al. 2021)
Walnut hydro‐ethanolic hull extract	Male Wistar rats	300 mg/kg, 7 days, gavage	↑ Antioxidant status, total thiols, AChE ↓ MDA, AOPP, cerebral cortex histological changes	(Sharma et al. 2021)
WPH	Rats	—	↑ Expression of BDNF, PSD95, p‐CREB ↓ Learning and memory deficits, inflammation in brain tissues, damaged hippocampal synaptic plasticity	(Xu and Zhao 2022)
Walnut kernel extract and juglone	Rats	Walnut kernel extract: 300 mg/kg, 21 days	↑ Brain dopamine levels ↓ Oxidative stress in liver and brain tissues	(Cintesun et al. 2023)
Walnut kernel methanolic extract	Female Wistar rats	50, 100 mg/kg, a week, gavage	↑ GSH level in BALF, GR and CAT levels in lung tissue ↓ LDH, total protein, total cell count in BALF, XO activity in lung tissue	(Qamar and Sultana 2011)
Walnut polyphenol fraction	C57BL/KsJ‐db/db mice	200 mg/kg, 4 weeks, p.o.	↓ Urinary 8‐hydroxy‐2′‐deoxyguanosin	(Fukuda et al. 2004)
Walnut leaves cyclohexane extract	Male Sprague Dawley rats	150, 250 mg/kg, 28 days, gavage	↑ HDL‐C ↓ FBG, LDL‐C, TC, TG, VLDL‐C, homocysteine, MDA amounts	(Jelodar et al. 2020)
Walnut hydro‐ethanolic extract	Male Wistar rats	0.05, 0.1, 0.2, 0.4 g/kg, 28 days, gavage	↑ SOD, CAT ↓ AST, ALT, ALP, fatty degeneration, necrosis, cytoplasmic vacuolization	(Eidi et al. 2013)
Walnut	Wistar albino rats	5, 10% of diet, 50 days, p.o.	↓ Serum ALT, GGT, LDH, MDA	(Bati et al. 2015)
Walnut	Male C57BL/6J mice	21.5% energy‐derived, 6 weeks, p.o.	↑SIRT 1, p‐AMPK/AMPK ↓ Hepatic lipid peroxidation, nitrated proteins, cytochrome P450‐2E1 levels, FAS, apoptosis	(Choi, Abdelmegeed, and Song 2016)
Walnut oligopeptides	Male Sprague Dawley rats	200, 440, 880 mg/kg, 30 days, gavage	↑ Antioxidant capacity, hepatic ethanol metabolizing enzymes ↓ Intoxication degree, blood ethanol concentration, hepatic steatosis, lipid oxidation products, expression of NF‐κB p65 expression in the liver	(Liu et al. 2023)
Walnut, walnut extract	Sprague Dawley rats		↑ SOD, CAT, renal and hepatic tissues structural integrity ↓ ALP, AST, dual‐oxidases expression	(Javed et al. 2022)
Clinical trial				
Walnut	15 healthy obese/overweight individuals + moderate hypercholesterolemia	Whole walnut: 85 g, Separated walnut skins: 5.6 g de‐fatted nutmeat: 34 g walnut oil: 51 g	↑ Cholesterol efflux ↓ RHI	(Berryman et al. 2013)
Walnut kernels	16 healthy participants	90 g	↑ Plasma γ‐tocopherol, (gallocatechin gallate, epicatechin gallate, epicallocatechin gallate, urine excretion of urolithin‐A in urine ↓ MDA, oxidized LDL)	(Haddad et al. 2014)

Abbreviations: ACh, acetylcholine; AChE, acetylcholinesterase; ALP, alkaline phosphatase; ALT, alanine transaminase; AMPK, adenosine monophosphate‐activated protein kinase; AOPP, advanced oxidation protein products; AST, aspartate transaminase; BALF, bronchoalveolar lavage fluid; BDNF, brain‐derived neurotrophic factor; BV‐2, bone marrow‐derived microglial cell line 2; cAMP, cyclic adenosine monophosphate; CAT, catalase; DNA, deoxyribonucleic acid; FAS, fatty acid synthase; FBG, fasting blood glucose; GGT, gamma‐glutamyl transferase; GR, glutathione reductase; GSH, glutathione; GSH‐Px, glutathione peroxidase; HDL‐C, high‐density lipoprotein cholesterol; HO‐1, heme oxygenase‐1; HT‐22, hippocampal neuronal cell line 22; JNK, Jun N‐terminal kinase; LDH, lactate dehydrogenase; LDL, low‐density lipoprotein; LDL‐C, low‐density lipoprotein cholesterol; MAPKs, mitogen‐activated protein kinases; MDA, malondialdehyde; MMP, matrix metalloproteinase; NF‐κB, nuclear factor kappa B; NO, nitric oxide; Nrf2, nuclear factor erythroid 2‐related factor 2; p‐AMPK, phosphorylated adenosine monophosphate‐activated protein kinase; PC12, Pheochromocytoma 12; p‐CREB, phosphorylated cAMP response element‐binding protein; PINK1, PTEN‐induced kinase 1; PKA, protein kinase A; PSD95, postsynaptic density protein; Ref, references; RHI, reactive hyperemia index; ROS, reactive oxygen species; SIRT1, silent information regulator 1; SNAP‐25, synaptosomal‐associated protein 25; SOD, superoxide dismutase; TC, total cholesterol; TG, triglycerides; VLDL‐C, very low‐density lipoprotein cholesterol; WEE, walnut ethanolic extract; WME, Walnut methanolic extract; WPH, walnut protein hydrolysate; WSE, walnut septum extract; XO, xanthine oxidase.

Three *J. regia* L. peptides—GGW, VYY, and LLPF [The full forms of these abbreviations were not explicitly stated in the paper. Typically, such abbreviations represent the amino acid sequences of the peptides, where each letter corresponds to a specific amino acid (e.g., G for Glycine, V for Valine, L for Leucine, W for Tryptophan, P for Proline, F for Phenylalanine, and Y for Tyrosine)]—were tested for their neuroprotective properties on PC12 cells that had been exposed to glutamate. Their potent radical scavenging activity, capacity to lower ROS generation, and ability to prevent superoxide dismutase (SOD) and glutathione peroxidase (GSH‐Px) depletion in PC12 cells may be the mechanism underlying the protective effects of GGW and VYY. LLPF's notable neuroprotective advantages can be mainly attributed to its potent inhibition of Ca^2+^ influx and mitochondrial membrane potential (MMP) collapse, even though it did not exhibit any obvious free radical scavenging activity in vitro. Furthermore, each of these peptides has the ability to control the production of proteins (B‐cell lymphoma 2 (Bcl‐2) and Bcl‐2‐associated protein × (Bax)) linked to apoptosis (Wang et al. 2018).

It has also been illustrated that treating LPS‐activated bone marrow‐derived microglial cell line 2 (BV‐2) microglia with WEKPPVSH, walnut peptide, increased SOD and catalase (CAT) activity, as well as the expression of nuclear factor erythroid 2‐related factor 2 (Nrf2) and heme oxygenase‐1 (HO‐1). The peptide attenuated ROS production, too (Gao et al. 2021).

An investigation was conducted to evaluate TW‐7, a polypeptide derived from *J. regia* L., in hippocampal neuronal cell line 22 (HT‐22) cells subjected to oxidative stress. TW‐7 prevented mitochondrial bioenergetic deficits, reduced H_2_O_2_‐induced opening of mitochondrial permeability transition pores, and restored the fluorescence intensity of the MMP. It substantially raised the SOD, GSH‐Px, and CAT activities. It reduced ROS levels in mitochondria and cells. In H_2_O_2_‐induced HT‐22 cells treated with Jun N‐terminal kinase (JNK) activator (anisomycin) and inhibitor (SP600125), TW‐7 also stimulated PTEN‐induced putative kinase 1 (PINK1)‐mediated mitophagy and downregulated JNK phosphorylation. Moreover, TW‐7 suppressed the production of cleaved caspase‐3, caspase‐9, and cytoplasmic cytochrome C, thereby inhibiting the mitochondrial apoptotic pathway. Moreover, synaptosomal‐associated protein of 25 (SNAP‐25) and brain‐derived neurotrophic factor (BDNF) levels rose considerably to protect synaptic function (Yang et al. 2022).

Furthermore, it has been observed that pretreating human neuroblastoma (SH‐SY5Y) cells with SGGY [Serine‐Glycine–Glycine‐Tyrosine], a novel tetrapeptide from walnut, before being exposed to H_2_O_2_ reduced oxidative stress and increased cell viability. It also enhanced nuclear translocation of Nrf2 and attenuated the levels of ROS and malondialdehyde (MDA), loss of MMP, apoptosis, as well as activation of JNK and p38 mitogen‐activated protein kinases (MAPKs) (Feng et al. 2023).

Treating SH‐SY5Y cells exposed to H_2_O_2_ with walnut polyphenols and urolithin A increased cell viability, the leakage of extracellular LDH, the activity of SOD, CAT, cyclic adenosine monophosphate (cAMP)‐dependent protein kinase A (PKA), expression of phosphorylated‐cAMP‐response element binding protein (p‐CREB), and BDNF. They also reduced apoptosis, intracellular calcium, and ROS production (An et al. 2023). In addition, walnut oil could ameliorate the in vitro Alzheimer's disease model on SH‐SY5Y cells by increasing acetylcholine (ACh) level and decreasing oxidative stress, protein oxidation, tau protein levels, and acetylcholinesterase (AChE) activity (Demirel et al. 2024).

##### 3.1.1.2 Monocytes

Treating human monocytic cell line (U937) cultured under hyperglycemic conditions with walnut oil exhibited a notable increase in the antioxidant capacity of cells and SOD activity. However, it did not influence cell proliferation and did not provide any protective effect against oxidative damage to DNA and proteins. Interestingly, the effects of walnut oil on inflammation were dual in nature, displaying both anti‐inflammatory and pro‐inflammatory effects depending on the duration of its incubation and its concentration (Laubertová et al. 2015).

##### 3.1.1.3 Keratinocytes

Treating human keratinocyte cell lines exposed to tumor necrosis factor‐alpha (TNF‐α) or t‐butyl hydroperoxide with *J. regia* L. ethanolic extract caused an increase in glutathione (GSH) amounts and a decrease in ROS production, lipid peroxidation, and the inflammatory process (Calcabrini et al. 2017).

#### 3.1.2 In Vivo

##### 3.1.2.1 Nervous System Disorders

It has been reported that oral administration of *J. regia* L. protein hydrolysates and its low molecular weight fraction to rats with memory deficits induced by sleep deprivation improved their behavioral performance, increased the amounts of SOD, GSH‐Px, CAT, and lowered MDA levels in brain tissue (Wang et al. 2018). In a transgenic model of Alzheimer's Disease in mice, adding walnut to the diet of animals could successfully enhance the activity of antioxidant enzymes and decline the levels of ROS, protein oxidation, and lipid peroxidation (Pandareesh et al. 2018). The supplementation of walnut suspension to rats with scopolamine‐induced memory impairment ameliorated memory function through increasing SOD, CAT, GSH‐Px, and lowering MDA levels in brain tissue (Haider et al. 2018) (Table 1).

The administration of walnut peptides (walnut protein hydrolysate and its low‐molecular‐weight fraction) to mice with memory deficit resulted in ameliorated cognitive impairment through reducing oxidative stress (increasing SOD and CAT activity besides lowering MDA amounts) and inflammatory responses in the brain (Wang et al. 2020). The antioxidant effects of walnut kernel and walnut septum extract in both a D‐galactose‐induced aging model and a naturally aged rat model were investigated. Rats were treated with D‐galactose, while older rats received walnut kernel or walnut septum extract supplementation. After 8 weeks, significant improvements were observed in cellular antioxidant activity, with reductions in ROS, advanced glycation end products, nitric oxide (NO), and MDA, alongside increased GSH levels. Both walnut kernel and walnut septum extract also lowered AChE activity in the brain. Histological and immunohistochemical analyses indicated that both supplements could protect neurons from senescence and reverse age‐related pathological conditions (Rusu et al. 2020).

Likewise, walnut protein hydrolysates could ameliorate behavioral performance, increase the expression of Nrf2, BDNF, CREB, levels of ACh, and ACh receptor in mice with amnesia. It also lessened AChE activity (Wang, Su, et al. 2021).

Pretreating rats with walnut hydro‐ethanolic hull extract before exposing them to isoprenaline boosted antioxidant status, total thiols, and AchE in rats' brains. It also lessened MDA, advanced oxidation protein product (AOPP), and cerebral cortex histological changes (Sharma et al. 2021). Another study evaluated the effect of walnut protein hydrolysate on rats with alcohol‐induced cognitive impairment. The substantial amelioration effect of walnut protein hydrolysate on learning and memory impairments was confirmed by the Morris water maze task. In addition, there was a notable decrease in oxidative stress in the brain tissues, and there was a remarkable upregulation of p‐CREB, BDNF, and postsynaptic density protein (PSD95) expression, which suggested that the impaired hippocampal synaptic plasticity had been restored. Furthermore, following the administration of walnut protein hydrolysate, there was a dose‐dependent improvement in the histopathological impairment in the rats' hippocampus, abnormal release of the neurotransmitters ACh and gamma‐aminobutyric acid (GABA), and disorders of the extracellular signal‐regulated kinases (ERK) and caspase‐3 signal pathways (Xu and Zhao 2022).

Moreover, it has been shown that the supplementation of walnut kernel extract and juglone increased brain dopamine levels and declined oxidative stress in liver and brain tissues compared to the serum in rats (Cintesun et al. 2023).

##### 3.1.2.2 Lung Injury

It has been shown that treating rats with cigarette smoke extract‐induced lung injury with walnut kernel methanolic extract enhanced GSH level in broncho‐alveolar lavage fluid (BALF) and elevated glutathione reductase (GR) and CAT levels in lung tissue. The extract also lowered LDH activity, total protein, total cell count in BALF, as well as xanthine oxidase (XO) activity in lung tissue (Qamar and Sultana 2011).

##### 3.1.2.3 Diabetes

Oral supplementation of *J. regia* L. polyphenol fraction to mice with type‐2 diabetes reduced urinary 8‐hydroxy‐2′‐deoxyguanosine, which is an oxidative stress marker (Fukuda et al. 2004). Walnut leaves cyclohexane extract was administered to streptozotocin‐induced diabetic rats, and it was observed that the extract could dose‐dependently increase high‐density lipoprotein cholesterol (HDL‐C) and decrease fasting blood glucose (FBG), low‐density lipoprotein cholesterol (LDL‐C), total cholesterol (TC), triglyceride (TG), very‐low‐density lipoprotein cholesterol (VLDL‐C), homocysteine, besides MDA amounts (Jelodar et al. 2020).

##### 3.1.2.4 Hepatotoxicity

Walnut hydro‐ethanolic extract revealed its hepatoprotective properties by augmenting SOD and CAT levels and decreasing aspartate aminotransferase (AST), alanine transaminase (ALT), alkaline phosphatase (ALP), fatty degeneration, necrosis, and cytoplasmic vacuolization in CCl_4_‐induced oxidative damage in rats (Eidi et al. 2013). Adding *J. regia* L. to the diet of rats on the experimental ethanol‐induced oxidative stress model decreased the levels of serum ALT, gamma‐glutamyl transferase (GGT), LDH, as well as MDA amounts in serum and other tissues (Bati et al. 2015).

The hepatoprotective effects of *J. regia* L. were examined on mice feeding with a high‐fat diet or rodent chow. By preventing high‐fat diet‐mediated changes to the levels of important proteins involved in lipid homeostasis, including fatty acid synthase (FAS), AMP‐activated protein kinase (AMPK), and sirtuin‐1 (SIRT1), walnut supplementation reduced the accumulation of fat. Furthermore, the hepatic levels of nitrated proteins, lipid peroxidation, and cytochrome P450‐2E1 were all markedly reduced. Additionally, walnuts reduced the p‐JNK and p‐p38K associated with activated cell death, which was accompanied by an increase in hepatocyte apoptosis in the high‐fat diet group (Choi, Abdelmegeed, and Song 2016).

The administration of walnut oligopeptides to rats with alcohol‐induced acute liver injury augmented antioxidant capacity, hepatic ethanol metabolizing enzymes, attenuated intoxication degree, blood ethanol amount, hepatic steatosis, lipid oxidation products, and expression of nuclear factor kappa B (NF‐κB) p65 in the liver (Liu et al. 2023).

##### 3.1.2.5 Arthritis

In arthritis‐induced oxidative stress model in rats the supplementation of *J. regia* L. or its extract resulted in increased levels of SOD, CAT, and renal and hepatic tissues structural integrity. Moreover, they attenuated ALP, AST amounts, as well as dual‐oxidases expression (Javed et al. 2022).

#### 3.1.3 Clinical Trial

##### 3.1.3.1 Cardiovascular System

A clinical trial examined the effect of acute consumption of de‐fatted walnut meat, walnut oil, separated walnut skins, and whole walnuts on postprandial endothelial function, lipemia, and oxidative stress in healthy overweight/obese individuals with moderate hypercholesterolemia. There was a significant interaction between the treatment and time point for TGs, indicating that different treatments had varying effects at different time points. Increased postprandial concentrations of TGs were observed specifically in the oil and whole walnut treatments. The presence of walnut skins resulted in a decrease in the reactive hyperemia index (RHI) compared to the baseline, and this difference continued to be evident when compared to the oil treatment. The oil treatment maintained the Framingham RHI compared to both the *J. regia* L. skins and the whole nut treatment. Additionally, there was a significant treatment effect on the ferric reducing antioxidant potential (FRAP), with higher mean FRAP values observed in the oil and skin treatments compared to the nutmeat treatment. In J774 cells cultured with postprandial serum, consuming whole walnuts led to an increase in cholesterol efflux compared to the fasting baseline. *J. regia* L. oil had a positive impact on endothelial function, while whole walnuts increased cholesterol efflux (Berryman et al. 2013).

A study aimed to determine whether the beneficial components of walnuts, such as ellagitannins and tocopherols, are easily absorbed by the human body and provide antioxidant protection following a meal. The researchers conducted a controlled feeding study on healthy people. Participants were given either a *J. regia* L. test meal or a meal produced with refined foods, with a minimum of 1 week between sessions. Blood samples were collected prior to meals and at intervals up to 24 h after intake to assess antioxidant levels and oxidative markers. The study found that walnut meal affected plasma γ‐tocopherol levels, but not α‐tocopherol levels. Over a 5‐h period after consuming the walnut meal, oxidative stress markers such as MDA decreased. Furthermore, both hydrophilic and lipophilic oxygen radical absorbance capacity rose following the walnut meal. The levels of total phenols, FRAP, and uric acid were similar between the two meals. At 2 h after eating the walnut meal, oxidized low‐density lipoproteins (LDL) levels reduced. Furthermore, 1 h after consuming food, the walnut meal raised plasma concentrations of certain catechins, including gallocatechin gallate, epicatechin gallate, and epicallocatechin gallate. The excretion of urolithin‐A, a beneficial component produced by ellagitannins, was likewise considerably higher in urine following the walnut meal (Haddad et al. 2014).

**FIGURE 1:**
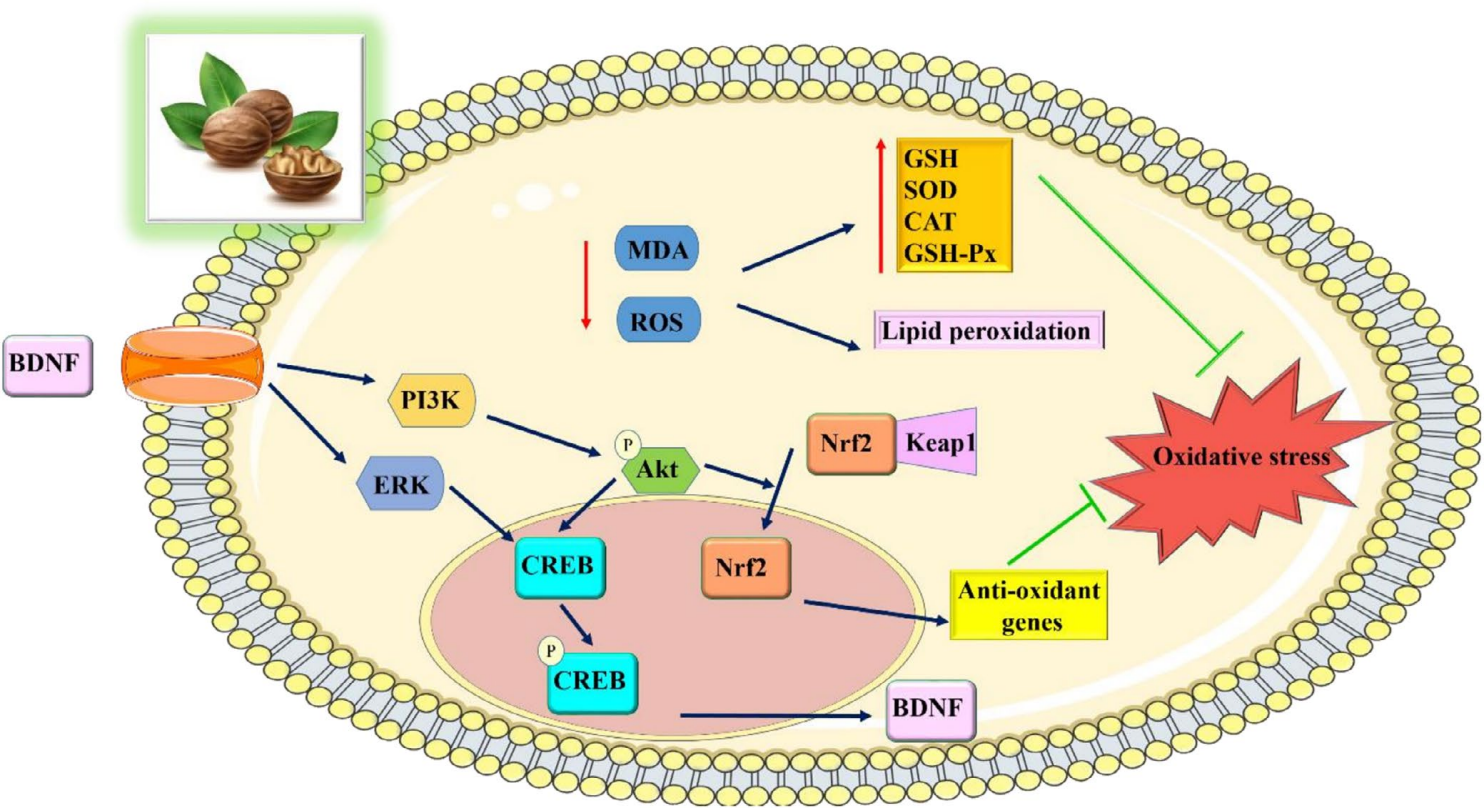
The proposed mechanism of antioxidant effects of *J. regia* L. (Images from https://smart.servier.com).

Taken together regarding the antioxidant effect of *J. regia* L., it could be stated that the antioxidant properties of *J. regia* L. (with the focus mostly on various walnut extracts, walnut kernels, and walnut peptides) have been extensively studied through in vitro, in vivo, and clinical trials. In vitro studies have demonstrated the ability of walnut extracts and compounds to protect various cell types, including neurons, monocytes, and keratinocytes, from oxidative damage. These protective effects are attributed to the scavenging of ROS, prevention of DNA damage, and maintenance of cellular redox balance. In vivo studies have confirmed the antioxidant effects of walnuts. These studies show that walnuts have antioxidant properties and could be used to treat a variety of ailments, including nervous system disorders, lung injury, diabetes, hepatotoxicity, and arthritis. The mechanisms underlying the antioxidant effects of walnuts involve the modulation of antioxidant enzyme activity, reduction of ROS levels, inhibition of protein oxidation, lipid peroxidation, and attenuation of oxidative stress‐induced damage (Figure 1).

The clinical trials shed light on the mechanisms of the antioxidant effects of walnut and its potential therapeutic applications in managing cardiovascular disorders. The studies examined the impact of acute consumption of different walnut components, including de‐fatted walnut meat, walnut oil, separated walnut skins, and whole walnuts, on postprandial endothelial function, lipemia, and oxidative stress. The results of these clinical trials illustrate that walnut consumption may have protective effects on the cardiovascular system and provide antioxidant protection. However, it is important to consider the limitations of these studies. The trials were conducted on specific populations, and further research is needed to determine the generalizability of the findings to broader populations. Additionally, the optimal dosage and duration of walnut consumption for cardiovascular health require further investigation.

While these studies demonstrate the antioxidant and therapeutic effects of walnut, it is important to note some limitations. Most of the studies discussed here were conducted on animal models, and the translation of these findings to human health needs further investigation. Moreover, the specific bioactive compounds responsible for the observed effects of walnut have not been fully elucidated. Further research is required to identify and understand the mechanisms of action of these compounds. Additionally, standardized dosages and long‐term safety studies are necessary to establish the optimal and safe use of walnut as a therapeutic agent in humans. Table 1 summarizes antioxidant properties of *J. regia* L. and its derivative.

### 3.2 Anti‐Inflammatory Effects of Walnut and Its Main Components

Inflammation is an innate immune system reaction that can be initiated by various elements, such as damaged cells, harmful substances, and pathogens (Chen et al. 2018). These elements have the potential to induce acute or chronic inflammatory responses in multiple organs and systems, including the heart (Ghasemzadeh Rahbardar et al. 2024), liver (Vafaeipour et al. 2023), kidney (Naraki et al. 2024), and brain (Parsi et al. 2024; Yin et al. 2022), which may result in tissue damage or illness. Inflammatory cells are activated and inflammatory signaling pathways, specifically the NF‐κB, MAPK, and the Janus kinase/signal transducers and activators of transcription (JAK/STAT) pathways, are triggered by both infectious and non‐infectious agents, as well as cellular damage (Mohammadi Zonouz et al. 2024).

Anti‐inflammatory agents are medications or substances that reduce inflammation in the body. They inhibit inflammatory cells and signaling pathways such as NF‐κB, MAPK, and JAK–STAT. These medicines are essential for controlling a variety of inflammatory disorders and relieving inflammation‐related symptoms (Shin et al. 2020; Zhao et al. 2021). Extensive studies have been conducted to explore the anti‐inflammatory characteristics of *J. regia* L. and its primary constituents, which will be discussed in the following section.

#### 3.2.1 In Vitro

##### 3.2.1.1 Neurons

The anti‐inflammatory protective effect of the peptide WEKPPVSH from walnut protein hydrolyzate on LPS‐activated BV‐2 microglia was investigated, as well as its potential mechanism. The findings showed that WEKPPVSH markedly and dose‐dependently reduced NO. Significant reductions in TNF‐α, interleukin (IL)‐1β, and IL‐6 secretion were observed with WEKPPVSH. Moreover, it inhibited the translocation of p65 to the cell nucleus. The expression of cyclooxygenase‐2 (COX‐2), p‐(NF‐kappa‐B inhibitor) IĸB/IĸB, pp65/p65, p‐p38/p38, and inducible nitric oxide synthase (iNOS) was down‐regulated by WEKPPVSH (Gao et al. 2021).

A study examined how the ethyl acetate fraction from walnuts, namely the Gimcheon 1 ho (EFGC) cultivar, protected hippocampus HT‐22 cells against particulate matter 2.5‐induced cytotoxicity. Significant FRAP, 1,1‐diphenyl‐2‐picrylhydrazl, and 3‐ethylbenzothiazoline‐6‐sulfonic acid radical scavenging functions were demonstrated by EFGC. The EFGC exhibited an inhibition of AChE activity and MDA production. Intracellular ROS level and cellular death of hippocampal cells were successfully reduced by the EFGC in response to particulate matter 2.5 cytotoxicity. By controlling the expression of proteins such as Bcl‐2, p‐JNK, Bax, caspase‐1, and p‐NF‐κB, the EFGC ameliorated inflammatory response and apoptosis (Moon, Kim, Lee, Kim, et al. 2022). Furthermore, the interaction between ellagic acid and a bioactive tripeptide from walnut meal was examined for its potential anti‐inflammatory properties. It was discovered that the interaction between ellagic acid and bioactive tripeptide greatly suppressed the LPS‐induced production of NO, TNF‐α, IL‐6, and IL‐1β in RAW264.7 macrophage cells (Cheng et al. 2022).

Another research examined the anti‐inflammatory mechanism of nanoparticles composed of ellagic acid and peptides from walnut meal. The treatment with nanoparticles resulted in a significant reduction in NO release. Furthermore, after treating RAW264.7 cells with nanoparticles, the results showed a substantial drop in iNOS messenger ribonucleic acid (mRNA) expression. Furthermore, data revealed that the nanoparticles blocked the NF‐κB upstream signaling pathway and altered the MAPK signaling pathway, which lowered the expression of inflammatory markers (Ling et al. 2023).

##### 3.2.1.2 Immune System Cells

The effects of *J. regia* L. oil on U937 cells under hyperglycemic conditions were found to be both anti‐inflammatory and pro‐inflammatory, depending on the oil concentration and incubation time. For a 2‐h incubation, all concentrations of walnut oil, except the highest, were effective at preventing the release of TNF‐α. After 16 h of incubation, all concentrations of the oil inhibited TNF‐α release; however, only the two lowest concentrations had an anti‐inflammatory effect after 24 h. Interestingly, the two lowest doses of walnut oil had a pro‐inflammatory effect, substantially raising the release of IL‐6. However, walnut oil had no effect on IL‐8 release at any concentration or incubation time. Furthermore, the two lowest concentrations of *J. regia* L. oil were able to suppress the release of monocyte chemoattractant protein‐1 (MCP‐1) after a 2‐h incubation, but this preventive effect was not observed after 16 and 24 h (Laubertová et al. 2015). Treating Raw 264.7 cells with walnut peptide could ameliorate the inflammatory response triggered by LPS through preventing M1 polarization (Guo et al. 2024).

##### 3.2.1.3 Red Blood Cells

It has been reported that treating human red blood cells with *J. regia* L. leaf methanolic extract stabilized cells against heat‐induced membrane lysis, as an indicator of anti‐inflammatory function (Polat et al. 2019). Moreover, the leaf extract of walnut, when tested at various concentrations, exhibited notable properties related to membrane stability and lipoxygenase inhibition on red blood cells. The membrane stability activity of the leaf extract was observed at a concentration of 800 μg/mL, which was found to be lower than that of ibuprofen. Additionally, at a concentration of 50 μg/mL, the leaf extract of walnut demonstrated higher lipoxygenase inhibitory activity compared to ibuprofen. Both the extract and ibuprofen displayed similar anti‐inflammatory activity at concentrations of 100, 200, and 400 μg/mL. However, at a concentration of 800 μg/mL, ibuprofen exhibited greater anti‐inflammatory activity compared to the leaf extract (Adebayo et al. 2024).

##### 3.2.1.4 Endothelial Cells

The effect of *J. regia* L. methanolic extract and ellagic acid, one of its major polyphenolic components, on the expression of vascular cell adhesion molecule (VCAM)‐1 and intracellular adhesion molecule (ICAM)‐1 in human aortic endothelial cells was evaluated because the expression of adhesion molecules by endothelial cells has been recognized as an early step in inflammation and atherogenesis. Ellagic acid and walnut extract both markedly reduced the TNF‐α‐induced endothelium production of ICAM‐1 and VCAM‐1 (Papoutsi et al. 2008).

##### 3.2.1.5 Fibroblasts and Keratinocytes

In an in vitro experiment with human keratinocytes exposed to TNF‐α or t‐butyl hydroperoxide, *J. regia* L. ethanolic extract treatment reduced inflammation‐related gene expression and showed anti‐inflammatory effects (Calcabrini et al. 2017).

Besides, preparing an aqueous extract of intact walnut seeds high in pellicles and assessing its anti‐inflammatory and anti‐aging qualities were the objectives of an in vitro study. The extract protected fibroblasts and keratinocytes against oxidative stress caused by an ultraviolet B (UVB) dose of 35 mJ/cm^2^ (5–6 h on a sunny day), preserved keratinocytes against UVB‐induced apoptotic death, restricted the development of inflammation in the epidermis, and inhibited elastase and collagenase (Przekora et al. 2018).

The methanolic extract of *J. regia* L. was found to have a photoprotective effect on human epidermal keratinocytes against inflammatory responses produced by ultraviolet B radiation. Significant free radical scavenging activity was identified in the methanolic extract of walnut, and it was shown to be comparable to that of standard antioxidants. Additionally, the methanolic extract of walnut has an 8.8 sun protection factor rating. Furthermore, in human epidermal keratinocytes, the methanolic extract of walnut pretreatment greatly lowered UVB‐activated inflammatory markers such as TNF‐α, IL‐1, IL‐6, NF‐κB, and COX‐2 (Muzaffer, Paul, Prasad, Karthikeyan, and Agilan 2018).

##### 3.2.1.6 Osteoblasts

The impact of *J. regia* L. methanolic extract on the formation of nodules in the osteoblastic cell line KS483 was evaluated and compared with ellagic acid. In KS483 osteoblasts, ellagic acid and walnut extract both promoted the development of nodules (Papoutsi et al. 2008).

##### 3.2.1.7 Colon Mucosal Epithelial Cells

The effects of the gastrointestinal digestion process simulation on the protein from *J. regia* L. and the possible anti‐inflammatory properties of its metabolites were investigated. Using molecular docking, three walnut polypeptides with strong anti‐inflammatory effects were discovered: FQGQLPR, VVYVLR, and IPAGTPVYLINR. It was shown that these polypeptides interacted with their target proteins in a flexible and stable manner. These peptides reduced vascular endothelial growth factor (VEGF), TNF‐α, and 5‐hydroxytryptamine (5‐HT) expression in normal human colon mucosal epithelial NCM460 cells exposed to LPS‐induced inflammation, while also reducing inflammation and cell apoptosis (Xia et al. 2024) (Table 2).

**TABLE 2:** The anti‐inflammatory effects of *J. regia* L. on in vitro, in vivo, and clinical trials.

Compound	Study design	Doses/duration	Results	Ref.
In vitro				
WEKPPVSH	BV‐2 microglia	12.5, 25, 50, 100, or 200 μM, 24 h	↓ NO, secretion of TNF‐α, IL‐1β, IL‐6, expression of COX‐2, p‐IĸB/IĸB, pp65/p65, p‐p38/p38, iNOS	(Gao et al. 2021)
EFGC	Hippocampal HT‐22 cells	—	‐ Regulated p‐JNK, p‐NF‐κB, Bcl‐2, Bax, caspase‐1 protein expression ↓ AChE activity, cellular death	(Moon, Kim, Lee, Kim, et al. 2022)
Walnut bioactive tripeptide and ellagic acid	RAW264.7 macrophage cells	—	↓NO, TNF‐α, IL‐6, IL‐1β production	(Cheng et al. 2022)
Walnut peptides and ellagic acid nanoparticles	RAW264.7 macrophage cells	—	↓ NO release, iNOS mRNA expression	(Ling et al. 2023)
Walnut oil	U937 cells	20, 40, 80, 159, 319 μg/mL, 2–24 h	‐Showed pro‐inflammatory and anti‐inflammatory effects	(Laubertová et al. 2015)
Walnut peptide	Raw 264.7 cells	0, 100, 200, 400, 800 μg/mL, 24 h	↓ Inflammatory response, M1 polarization	(Guo et al. 2024)
Walnut leaf methanolic extract	Human RBC	0.3959 mg/mL	↑ Membrane stabilization ↓ Heat induced lysis	(Polat et al. 2019)
Walnut leaf aqueous extract	RBC	50–800 μg/mL	↑ Membrane stabilization, lipoxygenase inhibitory activity	(Adebayo et al. 2024)
WME and ellagic acid	Human aortic endothelial cells	WME: 10–200 μg/mL Ellagic acid: 10^−7^–10^−5^ M	↓ ICAM‐1 and VCAM‐1 production	(Papoutsi et al. 2008)
WEE	Human keratinocytes cell line	—	↑ Anti‐inflammatory properties ↓ Expression of some genes involved in inflammatory process	(Calcabrini et al. 2017)
Undamaged walnut seeds aqueous extract	Fibroblasts, keratinocytes	2.5, 5, 10, 20, 40, 80, 160, 320, 640, 1280 μg/mL, 24, 48 h	‐ Preserved keratinocytes against UVB‐induced apoptotic death ↓ Inflammation in the epidermis, elastase, collagenase	(Przekora et al. 2018)
WME	Human epidermal keratinocytes	80 μg/ml	↑ Free radical scavenging activity ↓ROS production and lipid peroxidation, TNF‐α, IL‐1, IL‐6, NF‐κB, and COX‐2	(Muzaffer, Paul, Prasad, Karthikeyan, and Agilan 2018)
WME and ellagic acid	KS483 osteoblasts	WME: 10–50 μg/mL Ellagic acid: 10^−9^–10^−6^ M	↑ Development of nodules	(Papoutsi et al. 2008)
Walnut polypeptides	NCM460 cells	—	↓ 5‐HT, TNF‐α, VEGF expression, inflammation, cell apoptosis	(Xia et al. 2024)
In vivo				
Walnut	Male Fischer 344 rats	6, 9% of diet	↑ Autophagy in the hippocampus and striatum, ATG7, Beclin 1 ↓ Polyubiquitinated proteins aggregation, mTOR phosphorylation	(Poulose et al. 2013)
Glansreginin A	Male ICR mice	50, 100 mg/kg, 8 days, p.o.	↓ Abnormal behavior (immobility time), hyper‐activation of hippocampus microglia	(Haramiishi et al. 2020)
WEE	Male ICR mice	10, 20 mg/kg, 3 weeks, gavage	↑ Spatial learning, memory function, ACh levels, ChAT expression, MMP and ATP levels in brain tissue, ZO‐1, occludin, HO‐1, p‐AKT ↓ Mitochondrial function abnormality, AChE activity, TNF‐α, TNFR1, p‐JNK, IL‐1β, caspase‐3, oxidative stress	(Kim et al. 2020)
Walnut	Female NMRI mice	6% of diet	‐ No effect on IL1β gene expression ↑ HO levels ↓ Chronic inflammation	(Eckert et al. 2020)
Walnut kernels	Swiss albino mice	6, 9% of diet, 28 days, p.o.	↑ Water intake, locomotor activity, average rectal temperature ↓ Feed intake, immobility time, ptosis score, plasma corticosterone levels, brain nitrite levels	(Rani and Bansal 2021)
WPH	Rats	—	↑ Expression of BDNF, PSD95, p‐CREB ↓ Learning and memory deficits, oxidative stress, inflammation in brain tissues, damaged hippocampal synaptic plasticity	(Xu and Zhao 2022)
Walnut	Male Wistar rats	2.4 g, 6 weeks, p.o.	↓SIRT1/FoxO3a/MnSOD/CAT axis alterations in the heart, ChREBP nuclear fraction, systolic blood pressure, plasma AA/DHA and AA/EPA ratios	(Bošković et al. 2021)
Walnut septum acetone extract	Male Wistar rats	5, 10 mL, 3 days, gavage	↓ Cough frequency, IL‐6, CXC‐R1 amounts in lung, histopathological alterations	(Fizeșan et al. 2021)
Walnut kernel methanolic extract	Female Wistar rats	50, 100 mg/kg, a week, gavage	↓ LDH, total protein, total cell count in BALF, lung edema	(Qamar and Sultana 2011)
Walnut n‐hexane, methanol, and ethyl acetate extracts	BALB/c mice	500 mg/kg, 7 days, p.o.	↑ AQP‐1, AQP‐5 expression levels ↓ Airway inflammation, IL‐4, IL‐5, IL‐13, TNF‐α expression levels, inflammatory cells infiltration, goblet cells hyperplasia, TGF‐β, NF‐kB levels	(Sharif et al. 2022)
Walnut	Male C57BL/6J mice	21.5% energy‐derived, 20 weeks, p.o.	↑p‐AMPK in hepatic cells ↓ Hepatic TG, macrophage infiltration, pro‐inflammatory genes expression, apoptosis in adipose tissue, FAS in hepatic cells	(Choi, Abdelmegeed, Akbar, and Song 2016)
Walnut oil	Naive male C57BL/6 mice	7% of diet, 8 weeks, p.o.	↓ Damage score in inflamed tissue, changes in tight junction, free fatty acids receptors, pro‐inflammatory gene proteins expression	(Bartoszek et al. 2020)
Walnut	Male Sprague Dawley rats	2.80 g, 28 days, p.o.	↑Eicosapentaenoic acid and α‐linolenic acid levels in the liver and heart ↓12‐hydroxyeicosatetraenoic acid, 5‐F2t‐isoprostanes levels in liver	(Leung et al. 2021)
Walnut leaf hydroethanolic extract	Female C57BL/6 mice	2.5 g/kg, 17 days, p.o.	‐ No effect on total bacterial content ↑ IL‐10 ↓ *C. albicans* levels in the feces, expression of IL‐1β, TNF‐α in colon tissue	(Authier et al. 2022)
Leucine‐proline‐phenylalanine	Mice	100 mg/kg	↑ Colon length, abundance of Lachnospiraceae and Ruminococcaceae ↓ Colitis symptoms severity, colonic cell apoptosis, inflammatory factors production, regulatory T cells expansion	(Zhi et al. 2022)
Walnut oligopeptides	Male Sprague Dawley rats	200, 440, 880 mg/kg, 30 days, gavage	↑ Hepatic ethanol metabolizing enzymes ↓ Intoxication degree, blood ethanol concentration, hepatic steatosis, levels of pro‐inflammatory factors	(Liu et al. 2023)
Walnut green husk polysaccharides	Mice	—	↑ G6PC and PEPCK liver activity, amounts of Lactobacillus and Akkermansia, gut microbiota diversity ↓ TLR4/p65/IκBα pathway, oxidative stress, inflammatory markers expression	(Yang et al. 2023)
Walnut peptide	Male SPF‐grade BALB/c mice	500 mg/kg, 14 days, gavage	↑ Intestinal barrier integrity, mucus secretion, tight junction protein expression, abundance of beneficial bacteria ↓ Macrophage M1 polarization, NF‐κB signaling pathway, gut microbial disorder, abundance of harmful bacteria, IL‐6, IL‐1β, TNF‐α expression in colon tissue and serum	(Guo et al. 2024)
Walnut kernel powder	Broiler chickens	250, 500 mg/kg, 42 days, p.o.	↑ Growth, serum total protein, globulin, albumin, glucose, IgA, E, G ↓ Serum AST, ALT, creatinine, NF‐κB, IL‐6	(Oloruntola et al. 2024)
Walnut leaf ethanolic extract	Sprague Dawley rats	500 mg/kg, 12 days	↑ IL‐4 of blood ↓ Paw edema, arthritic development, blood levels of TNF‐α, NF‐κB, IL‐6, IL‐16, COX‐2, PGE2	(Mobashar et al. 2022)
Walnut root faction ointment	Wistar albino rats	1, 2.5, 5, 10% w/w	↑ Cutaneous wound healing ↓ Paw edema	(Huo et al. 2020)
Walnut green husk polysaccharides	Male Sprague Dawley rats	600 mg/kg, 50 days, gavage	↑ Colonic tight junction protein expression, gut microbiota dysbiosis, bacterial diversity in colon, browning of inguinal white adipose tissue and thermogenesis in the brown adipose tissue ↓ Weight gain, inflammation, oxidative stress, and colonic tissue damage, concentration of potentially harmful bacteria in colon	(Wang, Yang, et al. 2021)
Walnut peptide	Male C57BL/6J mice	220, 440, 880 mg/kg, 10 weeks, gavage	‐ Repaired intestinal mucosal barrier ↑ Glucose‐lipid metabolism ↓ Weight gain, hepatic lesions, inguinal adipose tissue adipocyte size, colonic tissue inflammatory infiltration, Firmicutes/Bacteroidetes ratio, intestinal inflammation	(Li et al. 2023)
Walnut seed coat ethanolic extract	Rats	150, 300 mg/kg, o.d.	↑ Aldose reductase, sorbitol dehydrogenase, butyrylcholinesterase activity ↓ AChE, GR, paraoxonase‐1, GST activity	(Palabıyık et al. 2023)
Walnut leaf aqueous and ethanolic extracts	Albino mice	Aqueous extract: 0.41, 1.64, 2.87 g/kg, 7 days, i.p. Ethanolic extract: 0.292, 1.17, 2.044 g/kg, 7 days, i.p.	↑ Anti‐inflammatory activity ↓Pain	(Hosseinzadeh et al. 2011)
Walnuts leaves aqueous extract and ointment	Male Swiss albino mice Male Californian rabbits	0.1, 0.5 g/mL	‐ Revealed analgesic, antispasmodic, anti‐inflammatory properties	(Amel et al. 2021)
Bioengineered walnut silver nanoparticles	Mice	—	↓ Cellular infiltrates, cytokine production, skin acute inflammation	(Awadallah and Hasan Al‐Nadaf 2023)
Walnut oil and kernel ethyl‐acetate extract	Male Albino mice	12.5, 25, 50 mg/kg, 8 days, gavage	↑ Serum CAT ↓ Lipid‐peroxidation, acute blood glucose level, neuropathic pain	(Raafat 2018)
Clinical trial				
Walnut	20 healthy men	—	↓TNF‐α mRNA expression, postprandial response in the mRNA of IL‐6 in peripheral blood mononuclear cells	(Jiménez‐Gómez et al. 2009)
Walnut oil extract	Active female students	2 g	↑ Cortisol, IL‐6	(Elahi and Atashak 2015)
Walnut	15 obese individuals with metabolic syndrome	48 g, 4 days, p.o.	↑ Serum apolipoprotein A	(Aronis et al. 2012)
Walnut	20 healthy trained elderly men	15 g, 6 weeks, p.o.	↑ HDL, testosterone ↓ TC, LDL, TG, serum cortisol, CRP	(Kamoun et al. 2021)

Abbreviations: 5‐HT, 5‐hydroxytryptamine; ACh, acetylcholinesterase; AQP, aquaporin; ATG7, autophagy‐related gene 7; BALF, broncho‐alveolar lavage fluid; Bax, Bcl‐2‐associated X protein; Bcl‐2, B‐cell lymphoma 2; BDNF, brain‐derived neurotrophic factor; BV‐2, bone marrow‐derived microglial cell line 2; CAT, catalase; ChREBP, carbohydrate‐responsive element‐binding protein; COX‐2, cyclooxygenase‐2; CREB, cAMP response element‐binding protein; CRP, C‐reactive protein; CXC‐R1, C‐X‐C motif chemokine receptor 1; FAS, fatty acid synthase; FOXO3a, transcription factor forkhead box O3 protein; G6PC, glucose‐6‐phosphatase; HDL, high‐density lipoprotein; HO‐1, heme oxygenase‐1; HT‐22, hippocampal neuronal cell line 22; ICAM, intracellular adhesion molecule; IL, interleukin; IκBα, inhibitor of nuclear factor kappa B; LDH, lactate dehydrogenase; LPS, lipopolysaccharide; MMP, mitochondrial membrane potential; MnSOD, manganese superoxide dismutase; mRNA, messenger ribonucleic acid; NF‐κB, nuclear factor kappa‐light‐chain‐enhancer of activated B cells; p65, nuclear factor kappa B subunit 65; p‐AKT, phosphorylated protein kinase B; p‐AMPK, phosphorylated 5′‐adenosine monophosphate‐activated protein kinase; PEPCK, phosphoenolpyruvate carboxyl kinase; PGE2, prostaglandin E2; p‐JNK, phosphorylated c‐Jun N‐terminal kinase; p‐mTOR, phosphorylated mammalian target of rapamycin; p‐p38, phosphorylated p38 mitogen‐activated protein kinase; PSD95, postsynaptic density protein; Ref, references; TGF‐β, transforming growth factor beta; TLR4, Toll‐like receptor 4; TNFR1, tumor necrosis factor receptor 1; VCAM, vascular cell adhesion molecule; VEGF, vascular endothelial growth factor; WEE, walnut ethanolic extract; WME, Walnut methanolic extract; WPH, walnut protein hydrolysate; ZO‐1, zonula occludens‐1.

#### 3.2.2 In Vivo

##### 3.2.2.1 Nervous System Disorders

Providing rats with a diet of walnuts decreased the amount of polyubiquitinated proteins that aggregated and triggered autophagy in the striatum and hippocampal regions. Animals fed walnuts showed increased autophagy through microtubule‐associated proteins 1A/1B light chain 3A (MAP1BLC3) protein turnover, mammalian target of rapamycin (mTOR) phosphorylation inhibition, and increased expression of autophagy‐related 7 (ATG7) and Beclin 1. The hippocampal region experienced a greater clearance of polyubiquitinated protein aggregates, such as sequestosome‐1 (p62/SQSTM1), compared to the striatum. The phosphorylation of cyclic AMP response element binding protein and NF‐κB, as well as the levels of P38‐MAP kinase, demonstrated that the clearing of ubiquitinated aggregates was accompanied by large reductions in oxidative stress/inflammation (Poulose et al. 2013). Moreover, in the LPS‐induced inflammatory mice model, the administration of glansreginin A, derived from aqueous‐methanolic extract of walnut, ameliorated abnormal behavior by decreasing immobility time and hyper‐activation of hippocampus microglia (Haramiishi et al. 2020).

Walnut ethanolic extract revealed an anti‐amnesic effect in mice by improving spatial learning and memory function through enhancing ACh levels, choline acetyltransferase (ChAT) expression, increasing MMP and adenosine triphosphate (ATP) levels in brain tissue, augmenting zonula occludens‐1 (ZO‐1), occludin, HO‐1, p‐protein kinase B (AKT) levels, decreasing mitochondrial function abnormality, AChE activity, the amounts of TNF‐α, tumor necrosis factor receptor 1 (TNFR1), p‐JNK, IL‐1β, caspase‐3, and oxidative stress (Kim et al. 2020).

Assessing the effect of *J. regia* L.‐enriched diet on aged mice regarding the oxylipin compounds involved in inflammation profile in the brain revealed that the walnut diet did not affect the expression of the IL‐1β gene in the brains of mice. The levels of oxylipins were generally higher in the brains of aged mice compared to young mice. When aged mice were fed a walnut diet, the levels of oxylipins derived from fatty acids specific to walnuts increased in the brain. Specifically, hydroxy‐oxylipins based on the fatty acid linoleic acid were significantly increased in the brains of walnut‐fed mice compared to control mice. On the other hand, hydroxy‐oxylipins based on α‐linolenic acid were detected in the brains of mice on the walnut diet (Eckert et al. 2020). Pre‐treating mice with *J. regia* L. kernels in an experimental model of depression enhanced their locomotor activity, average rectal temperature, and decreased immobility time, ptosis score, plasma corticosterone levels, as well as brain nitrite levels compared to the untreated animals (Rani and Bansal 2021).

In rats with alcohol‐induced cognitive impairment, the study evaluated the ameliorative impact of *J. regia* L. protein hydrolysate. The substantial amelioration effect of walnut protein hydrolysate on learning and memory impairments was confirmed by the Morris water maze task. Additionally, there was an apparent reduction in inflammation within the brain's tissues, and the noteworthy upregulation of BDNF, PSD95, and p‐CREB expression suggested that the impaired hippocampal synaptic plasticity was being recovered. Furthermore, following walnut protein hydrolysate administration, there was a dose‐dependent improvement in the histopathological impairment in the hippocampus of alcohol‐induced cognitive impairment rats, abnormal release of the neurotransmitters GABA and ACh, and alterations of the ERK and caspase‐3 signal pathways (Xu and Zhao 2022).

##### 3.2.2.2 Cardiovascular System Disorders

Walnut has been shown to have cardioprotective properties in rats with fructose‐induced increases in plasma predictors of low‐grade inflammation and decreases in antioxidative/anti‐inflammatory activity of cardiac tissue. *J. regia* L. reduced variations in the SIRT1/transcription factor forkhead box O3 protein (FOXO3a)/manganese superoxide dismutase (MnSOD)/CAT axis in the heart, carbohydrate response element‐binding protein (ChREBP) nuclear fraction, systolic blood pressure, plasma arachidonic acid/docosahexaenoic acid, and arachidonic acid/eicosapentaenoic acid ratios (Bošković et al. 2021).

##### 3.2.2.3 Lung Injury

The administration of *J. regia* L. kernel methanolic extract to rats with cigarette smoke extract induced acute inflammation and lung injury, decreased lung edema by lowering LDH, and attenuating total protein and total cell count in BALF (Qamar and Sultana 2011).

The supplementation of walnut septum acetone extract to rats exposed to citric acid aerosol reduced cough frequency, attenuated IL‐6, C‐X‐C motif chemokine receptor 1 (CXC‐R1) amounts in lung, and lessened histopathological alterations in lung tissue. The antitussive property of *J. regia* L. septum acetone extract was better than codeine (Fizeșan et al. 2021). The administration of walnut n‐hexane, methanol, and ethyl acetate extracts to asthmatic mice reduced airway inflammation by increasing aquaporin (AQP)‐1, AQP‐5 expression levels and attenuating IL‐4, IL‐5, IL‐13, TNF‐α expression levels, inflammatory cell infiltration, goblet cell hyperplasia, as well as transforming growth factor‐beta (TGF‐β), NF‐κB levels (Sharif et al. 2022).

##### 3.2.2.4 Digestive System Disorders

The administration of *J. regia* L. to high fat‐fed mice increased p‐AMPK and decreased FAS in hepatic cells. The results indicated that walnut lowered hepatic TG, macrophage infiltration, pro‐inflammatory genes expression, as well as apoptosis in adipose tissue. However, it had no significant effects on visceral fat mass, body weight, insulin resistance, and peripheral glucose intolerance (Choi, Abdelmegeed, Akbar, and Song 2016). In addition, walnut oil ameliorated intestinal inflammation induced by dextran sulfate sodium in mice through lowering the damage in inflamed tissue, reducing the changes in tight junction, free fatty acids receptors, and pro‐inflammatory gene proteins expression (Bartoszek et al. 2020).

Administration of *J. regia* L. enhanced the levels of eicosapentaenoic acid and α‐linolenic acid in the liver and heart tissues of rodents compared to the control animals. It also reduced the amounts of 12‐hydroxyeicosatetraenoic acid and 5‐F2t‐isoprostanes, triggered 4‐F4t‐neuroprostane, besides considerable levels of anti‐inflammatory hydroxydocosahexaenoic acid in the liver tissue (Leung et al. 2021).

The effect of *J. regia* L. leaf hydroethanolic extract on gastrointestinal colonization by *Candida albicans* , gut microbiota composition, and colon inflammation was assessed in mice. The results indicated that although the extract had no substantial effect on total bacterial content, it reduced *C. albicans* levels in the feces and decreased the expression of IL‐1β and TNF‐α in colon tissue. It also increased IL‐10 amounts in colon tissue (Authier et al. 2022).

On mice with colitis induced by dextran sulfate sodium, the advantages of the walnut‐derived peptide leucine‐proline‐phenylalanine were studied. The inflammatory cytokine levels of serum, disease activity index score, and changes in body weight all showed that leucine‐proline‐phenylalanine lessened the severity of symptoms during the colitis development phase. In addition, the treatment groups had beneficial results such as longer colons, lower colonic cell apoptosis, reduced inflammatory factor production, and greater splenic regulatory T cell expansion during the recovery phase. Additionally, leucine‐proline‐phenylalanine restored the dysbiosis of the gut microbiota in colitis mice, according to 16S ribosomal DNA sequencing examinations. This includes an increase in the relative abundance of the Ruminococcaceae and Lachnospiraceae families throughout the colitis development phase, as well as a partial recovery of microbiota diversity. Furthermore, during the colitis recovery period, the gut microbiota composition progressively improved, displaying a higher relative abundance of helpful genera and a lower abundance of possibly detrimental genera in comparison to the dextran sulfate sodium group (Zhi et al. 2022). It has been reported that walnut oligopeptides administration in rats with alcohol‐induced acute liver injury increased hepatic ethanol metabolizing enzymes, reduced hepatic steatosis, blood ethanol amount, and levels of pro‐inflammatory factors in the liver (Liu et al. 2023).

It has been discovered that walnut green husk polysaccharides exhibited remarkable protective properties against ochratoxin A‐induced hepatic inflammation and gluconeogenesis dysfunction in mice. First, in the liver, walnut green husk polysaccharides blocked the Toll‐like receptor 4 (TLR4)/p65/inhibitor of nuclear factor kappa B (IκBα) pathway, reduced oxidative stress, and down‐regulated the expression of inflammatory markers. Subsequently, *J. regia* L. green husk restored the decreased activity of glucose 6‐phosphatase (G6PC) and phosphoenolpyruvate carboxyl kinase (PEPCK) in the liver induced by ochratoxin A. Additionally, walnut green husk polysaccharides enhanced the amount of beneficial bacteria, including Lactobacillus and Akkermansia, as well as the diversity of the gut microbiota. The outcomes of the fecal microbiota transplantation experiment also provided additional evidence that the gut microbiota plays a role in the protective properties of walnut green husk polysaccharides against ochratoxin A‐induced liver injury (Yang et al. 2023).

Furthermore, it has been reported that the supplementation of *J. regia* L. peptide to mice with colitis increased intestinal barrier integrity, mucus secretion, tight junction protein expression, and the abundance of beneficial bacteria. It also reduced macrophage M1 polarization, NF‐κB signaling pathway, gut microbial disorder, the abundance of harmful bacteria, IL‐6, IL‐1β, and TNF‐α expression in colon tissue and serum (Guo et al. 2024).

Administration of walnut kernel powder in broiler chickens receiving aflatoxin in their diets increased their growth, serum levels of total protein, globulin, albumin, glucose, and immunoglobulin (Ig) A, E, G in comparison with the untreated birds. It also reduced serum amounts of AST, ALT, creatinine, NF‐κB, and IL‐6 (Oloruntola et al. 2024).

##### 3.2.2.5 Arthritis

Walnut leaf ethanolic extract ameliorated acute inflammation and arthritis in rats by increasing the levels of blood IL‐4, besides attenuating TNF‐α, NF‐κB, IL‐6, IL‐16, COX‐2, and prostaglandin E2 (PGE2) amounts in blood, which resulted in reduced paw edema and arthritic development (Mobashar et al. 2022).

##### 3.2.2.6 Skin Wounds

It has been reported that using walnut root faction ointment in rats speeded up the cutaneous wound healing and lowered paw edema (Huo et al. 2020).

##### 3.2.2.7 Obesity and Dyslipidemia

It has been investigated how *J. regia* L. green husk polysaccharides protect against obesity, oxidative stress, inflammation, liver damage, and colon damage in rats fed a high‐fat diet. It was discovered that walnut green husk polysaccharides improved the expression level of colonic tight junction protein in the rats and reduced the aberrant weight gain, inflammation, oxidative stress, and colonic tissue damage stimulated by high fat. Additionally, the walnut green husk polysaccharides enhanced gut microbiota dysbiosis by raising bacterial diversity and lowering the relative concentration of potentially harmful bacteria in the rats' colons. This resulted in the browning of inguinal white adipose tissue and thermogenesis in the brown adipose tissue of high‐fat‐fed rats. Consuming walnut green husk polysaccharides boosted the relative abundance of Prevotellaceae and Allobaculum in the rats' guts in addition to increasing the quantity of short‐chain fatty acids (Wang, Yang, et al. 2021).

It has been illustrated that *J. regia* L. peptide intervened in high‐fat diet‐induced dyslipidemia and obesity in mice by repairing the intestinal mucosal barrier, enhancing glucose‐lipid metabolism, reducing weight gain, hepatic lesions, inguinal adipose tissue adipocyte size, decreasing colonic tissue inflammatory infiltration, lowering the Firmicutes/Bacteroidetes ratio, as well as intestinal inflammation (Li et al. 2023).

Researchers investigated the potential amendatory effects of ethanol extract of walnut seed coat on the activity of several metabolic enzymes in the liver, kidney, and heart of rats that had hyperlipidemia induced by Triton WR‐1339. The activity of aldose reductase, sorbitol dehydrogenase, and butyrylcholinesterase enzymes was significantly elevated in all tissues in the Triton WR‐1339 group as compared to the control, while the activity of the other investigated enzymes (AChE, GR, paraoxonase‐1, glutathione S‐transferase (GST)) was substantially lowered. Ethanol extract of walnut seed coat avoided changes in the activity of the examined metabolic enzymes and lessened the effect of hyperlipidemia on balance (Palabıyık et al. 2023).

##### 3.2.2.8 Pain and Spasm

Walnut leaf aqueous and ethanolic extracts exhibited anti‐inflammatory property against chronic and acute inflammation as well as peripheral and central antinociceptive effects through non‐opioid receptors (Hosseinzadeh et al. 2011).

An in vivo study was designed to assess the healing property, antispasmodic, and anti‐inflammatory effects of *J. regia* L. leaf aqueous extracts and ointment. The analgesic effect was tested using the acetic acid‐induced endogenous spasm test, the healing effect was tested on rabbits, and the anti‐inflammatory activity was screened on mice using the carrageenan‐induced paw edema test, which *J. regia* L. aqueous extract showed a notable anti‐inflammatory effect. Comparing the analgesic effect results with those from the reference product Spasfon, it was found that they were statistically significant. Rabbits were used to test the healing effect, and the results showed a high degree of healing power when compared to those obtained with the pharmaceutical healing paste “MADICASSOL” as a reference drug. Comparing the ointment of reference, which took over 10 days to fully cure, to the one made with walnut leaves, the former has demonstrated greater efficacy, with a healing delay of less than a week (Amel et al. 2021).

Another study intended to determine whether the walnut fruit extract with acetonitrile had an anti‐inflammatory effect on an experimental animal model of inflammation. The formalin test was utilized in the study to evaluate the antinociceptive effects of carrageenan in mice, with rat paw edema used as an induction model for inflammation. While leaf extract has a stronger late anti‐inflammatory effect, the combination with unripe fruit bio‐reduced nanoparticles and extract exhibits a greater acute anti‐inflammatory effect. By lowering cellular infiltrates and cytokine production, these bioengineered nanoparticles effectively reduce acute inflammation of the skin (Awadallah and Hasan Al‐Nadaf 2023).

##### 3.2.2.9 Diabetes

The antidiabetic and antidiabetic‐neuropathy, and acute anti‐inflammatory properties of *J. regia* L. oil and kernel ethyl acetate extract were examined in mice. The greatest improvement in chronic blood glucose reduction, acute inflammatory pain, elevated serum insulin, and normalization of glycated hemoglobin levels has been demonstrated by linoleic acid. Higher lipid peroxidation decrease has been observed in walnut oil, although higher acute blood glucose reduction and serum CAT increase have been found in kernel ethyl acetate extract. The highest doses of linoleic acid, walnut oil, and kernel ethyl acetate extract have demonstrated stronger antinociceptive potentials in comparison to tramadol in the treatment of thermal‐hyperalgesic and anti‐allodynic neuropathic pain (Raafat 2018).

#### 3.2.3 Clinical Trials

##### 3.2.3.1 Inflammatory Response

A clinical trial assessed the effects of dietary fat on the postprandial expression of pro‐inflammatory genes in peripheral blood mononuclear cells of healthy participants, as inflammation is important in all stages of atherosclerosis. In peripheral blood mononuclear cells, the butter breakfast increased TNF‐α mRNA expression more than the walnut meal did. Furthermore, compared to the walnut breakfast, there was a greater postprandial response in the mRNA of IL‐6 in these cells when butter and olive oil were consumed. Nevertheless, there were no appreciable variations in the effects of fatty breakfasts on the plasma concentrations of these pro‐inflammatory markers (Jiménez‐Gómez et al. 2009). Moreover, the effect of consuming extracts from *J. regia* L. oil on markers of inflammation after a strenuous workout was investigated in active female students. The results demonstrated a significant increase in cortisol and IL‐6 levels in both groups and in all post‐activity phases (Elahi and Atashak 2015).

##### 3.2.3.2 Lipid Profile

The consumption of walnut in obese individuals with metabolic syndrome resulted in increased levels of serum apolipoprotein A. However, it had no significant effects on the amounts of amyloid A, C‐reactive protein (CRP), fetuin A, E‐selectin, IL‐6, IL‐8, P‐selectin, resistin, soluble vascular cell adhesion protein 1, soluble intercellular adhesion molecules 1 and 3, TNF‐α, and thrombomodulin (Aronis et al. 2012).

It has been shown that consuming moderate amounts of walnut improves lipid profile by enhancing HDL level and lowering TC, LDL, and TG amounts. It also increases testosterone and attenuates the levels of serum cortisol and CRP in trained elderly men (Kamoun et al. 2021) (Table 2).

**FIGURE 2:**
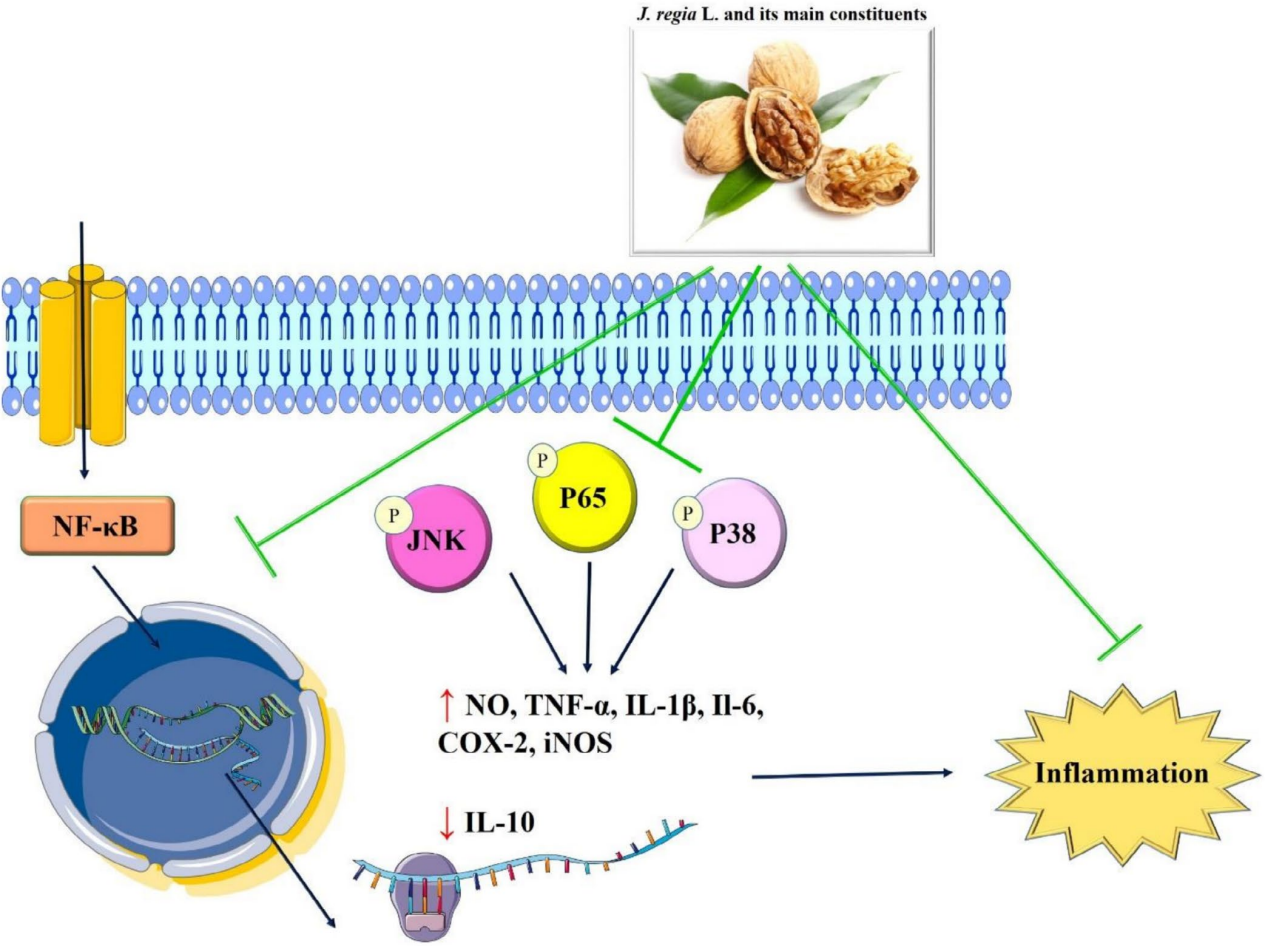
The suggested underlying mechanism of anti‐inflammatory properties of *J. regia* L. (Images from https://smart.servier.com).

Taken together regarding the anti‐inflammatory effect of *J. regia* L., it could be stated that the anti‐inflammatory properties of *J. regia* L. (with the focus mostly on various walnut extracts, walnut peptides, and walnut kernels) have been widely explored through in vitro, in vivo, and clinical investigations. The findings from in vitro studies underscore the potential of walnut‐derived components in modulating inflammatory responses across various cell types. Peptides, extracts, and compounds from *J. regia* L. demonstrated anti‐inflammatory effects by targeting key inflammatory mediators, signaling pathways, and cellular processes (Figure 2). The research on walnuts in various in vivo models reveals promising therapeutic effects on the nervous system, cardiovascular system, lung disorders, digestive system disorders, arthritis, skin wounds, obesity, dyslipidemia, pain, spasm, and diabetes. Studies suggest that walnuts may improve autophagy, cognitive function, and reduce inflammation and oxidative stress markers. Moreover, *J. regia* L. components such as walnut oil, walnut leaf hydroethanolic extract, walnut‐derived peptides, walnut green husk polysaccharides, and walnut peptides have shown favorable results in alleviating inflammation, improving metabolic profiles, modulating gut microbiota, and enhancing overall health outcomes in animal models. The strengths of these studies include the diverse effects of walnuts across different systems, mechanistic insights into their actions, and the use of multiple animal models. Also, these studies provide valuable insights into the potential therapeutic applications of walnut components in managing chronic conditions.

In clinical trials, walnut consumption improved lipid profiles by boosting HDL and reducing TC, LDL, and TG levels, as well as altering hormone levels like testosterone and lowering cortisol and CRP. These studies provided direct insights into the effects of walnut and its derivatives on inflammation and lipid profiles. The findings revealed specific responses in gene expression and lipid markers, contributing to a better understanding of the impact of walnut consumption on health parameters. The research included diverse participant groups, such as healthy individuals, obese individuals with metabolic syndrome, active female students, and trained elderly men, enhancing the generalizability of the results. However, the studies focused on short‐term effects, and long‐term implications of walnut consumption on inflammation and lipid profiles remained unclear. Variability in participant characteristics, such as age, health status, and fitness levels, could influence the outcomes, warranting further investigation for specific populations. Furthermore, the effect of *J. regia* L. dosage, duration of consumption, and potential interactions with other dietary components or medications were not extensively explored. Besides, additional factors like lifestyle habits, dietary patterns, and genetic predispositions were not thoroughly accounted for in the studies, potentially affecting the observed results.

By addressing these limitations and incorporating these suggestions in future research, a more comprehensive understanding of the effects of walnut consumption on inflammation and lipid profiles can be gained, potentially providing valuable insights for preventive and therapeutic interventions in various health conditions. Table 2 summarizes anti‐inflammatory properties of *J. regia* L. and its derivative.

### 3.3 Regulation of Apoptosis by Walnut and Its Main Components

Apoptosis, a controlled and predetermined process of cell death, is an essential mechanism for preserving tissue homeostasis. Numerous factors, such as cellular damage, external stresses, and certain signaling pathways, can cause apoptosis (Fernández‐Lázaro et al. 2024). Numerous disorders that affect essential organs like the heart (Gong et al. 2023), liver (Karimi et al. 2023), kidneys (Naraki et al. 2024), lungs (Ghasemzadeh Rahbardar et al. 2023), and brain (Ghobakhlou et al. 2023; Mohammadzadeh et al. 2022) which are associated with tissue damage and pathological states, have been linked to dysregulation of apoptosis.

A series of events known as apoptotic pathways are arranged by important regulators such as caspases and members of the Bcl‐2 family of proteins (Rajabian et al. 2023; Tandisehpanah et al. 2022). A disturbance in this balance may lead to either excessive mortality of cells or inadequate elimination of damaged cells, hence facilitating the advancement of the disease. Notably, a number of signaling pathways—such as the death receptor pathway and the mitochondrial pathway—are essential for activating and executing apoptosis in response to both internal and external stimuli (Singh et al. 2019).

Addressing the complex mechanisms involved in apoptosis and investigating potential interventions to modify apoptotic pathways are essential in developing targeted treatments for disorders marked by abnormal processes associated with cell death. Further research into the anti‐apoptotic properties of natural substances, like those in *J. regia* L., may provide novel ideas on treating illnesses associated with apoptosis and improving tissue health.

#### 3.3.1 In Vitro

##### 3.3.1.1 Promyeoloblasts

In HL‐60 cells, green husk ethanolic extract has an antiproliferative effect that is associated with the activation of early apoptosis and a loss of MMP. When juglone is present at 1 μM concentrations, neither malignant nor healthy cells are affected; but at higher concentrations, both types of cells lose viability (Soto‐Maldonado et al. 2019), (Table 3).

**TABLE 3:** The apoptosis regulatory effects of *J. regia* L. on in vitro and in vivo experiments.

Compound	Study design	Doses/duration	Results	Ref.
In vitro				
Walnut green husk polyphenolic extract	HL‐60 cells	1, 10 μM juglone	‐ No effect on normal fibroblast cells ↑ Antiproliferative effect, apoptosis ↓ MMP	(Soto‐Maldonado et al. 2019)
Walnut hulls phenolic extract	Bone tumor cells	0–1000 μg/mL	‐ Showed less cytotoxic properties on normal cells ↑ Caspase activity, apoptosis ↓ Cell proliferation, tumor cell migration, MMP, intracellular ROS	(Khani and Meshkini 2021)
Walnut green husk methanol, chloroform, and n‐hexane extracts	PC3 cells	0, 5, 10, 20, 40, 80, 160 μg/mL, 24 h	↑ Apoptosis, gene expression of Bax, caspase‐3, TP53 ↓ Cell proliferation, Bcl‐2	(Alshatwi et al. 2012)
Walnut leaf hexane extract	PC3 cells	5, 25, 50, 75, 100 μg/ml, 48 h	↑ Anti‐proliferative, apoptosis, apoptotic bodies, cells in the sub‐G1 phase ↓ Cells in the G2/M phase	(Li et al. 2015)
Urolithin A	LNCaP prostate cells	40 μM, 24 h	↑ CDKN1A expression, apoptotic cells, cell cycle arrest ↓ PDK1 expression	(Sanchez et al. 2015)
Walnut leaf chloroform extract	MCF‐7, BHY cells	10–80 μM	↑ Cleavage of caspase‐3 ↓ Proliferation of cells, MCF‐7 growth	(Salimi et al. 2014)
Walnut residual protein	MCF‐7, Caco‐2, HeLa, IEC‐6 cells	0.5–4 mg/mL	‐ No cytotoxic activity on IEC‐6 cells ↑ Apoptosis and autophagy on MCF‐7 cells, NO production in macrophages, IL‐2 secretion and proliferation in spleen lymphocytes ↓ MCF‐7, Caco‐2, HeLa growth	(Ma et al. 2015)
Urolithin A	MCF‐7 cells	40 μM, 24 h	↑ CDKN1A, PTEN expression, apoptotic cells, cell cycle arrest ↓ PDK1 expression	(Sanchez et al. 2015)
Walnut peptides‐zinc complex	Human breast carcinoma cells, MCF‐7 cells	37.25, 75, 150, 300 μg/mL, 48 h	↑ Antiproliferative activity, cell apoptosis, cell cycle arrest, ↓ MCF‐7 cells growth	(Liao et al. 2016)
Walnut milk	TG/HAVSMC, MCF7, Du145 cells	—	‐ No effect on noncancerous cells ↑ Intrinsic apoptotic signaling, ROS production, caspase‐dependent death, cell viability	(Doganlar and Doganlar 2016)
Walnut outer shells methanol, ethanol, and hexane extracts	MDA‐MB‐231 cells	0.125, 0.25, 0.5, 1, 2 mg/mL	↑ Cytotoxicity effects	(Dalkılıç et al. 2023)
Walnut raw and roasted fermentation supernatants	LT97 cells	2.5% and 5%, 24 h	↑ CAT and GSTT2 mRNA levels, early apoptotic cells, caspase‐3 activation ↓ GSH‐Px1 levels, cell proliferation early apoptotic cells	(Schlörmann et al. 2017)
Male walnut flower methanolic extract	HaCaT cells	80 μg/ml	↓ Apoptotic characteristics, DNA damage, decline in MMP	(Muzaffer, Paul, Prasad, and Karthikeyan 2018)
Green walnut husks	CT26 cells	—	↑ Apoptosis ↓ Tumor growth	(Chen et al. 2023)
In vivo				
Walnut oil	Female rats	0.6, 1.2, 2.4 g/kg, 9 days, i.g.	↑ Total SOD activity, Bcl‐2 levels ↓ MDA, Bax levels in hippocampal dentate gyrus cells, apoptosis	(Chen et al. 2011)
WEE	Male C57BL/6 mice	20, 50 mg/kg, 4 weeks, p.o.	↑ p‐AKT ↓ Behavioral and memory impairment serum dyslipidemia, liver fat mass, white adipose tissue, diabetic oxidative stress, cholinergic system impairment, ROS formation, Bax, caspase‐3, p‐JNK, brain damage	(Moon, Kim, Lee, Kang, et al. 2022)
Walnut oligopeptides	Female BALB/c mice	14 days, 0.22, 0.44, 0.88 g/kg p.o.	↑ Antioxidant defense system, hematopoietic recovery, survival rate, epithelial integrity, Bcl‐2, IĸB ↓ Weight loss, intestinal epithelial barrier dysfunction, promoting epithelial integrity, splenocyte apoptosis, Bax, caspase‐3, NF‐κB inflammatory cascade	(Zhu et al. 2019)

Abbreviations: Bax, Bcl‐2‐associated X protein; CAT, catalase; CDKN1A, cyclin‐dependent kinase inhibitor 1A; GSH‐Px1, glutathione peroxidase 1; GST, glutathione S‐transferase; GSTT2, glutathione S‐transferase theta 2; IL‐2, interleukin‐2; LNCaP, lymph node carcinoma of the prostate; MCF‐7, Michigan Cancer Foundation‐7; MDA, malondialdehyde; MMP, mitochondrial membrane potential; mRNA, messenger ribonucleic acid; NF‐κB, nuclear factor kappa‐light‐chain‐enhancer of activated B cells; p‐AKT, phosphorylated protein kinase B; PDK1, 3‐phosphoinositide‐dependent protein kinase‐1; p‐JNK, phosphorylated c‐Jun N‐terminal kinase; PTEN, phosphatase and tensin homolog; ROS, reactive oxygen species; SOD, superoxide dismutase; TP53, tumor protein p53, WEE, walnut ethanolic extract.

##### 3.3.1.2 Bone Tumor Cells

The anti‐proliferative effect of walnut hulls phenolic extract was investigated on bone tumor cells. The findings showed that the extract increased caspase activity and apoptosis. Moreover, it reduced cell proliferation, tumor cell migration, MMP, as well as intracellular ROS (Khani and Meshkini 2021).

##### 3.3.1.3 Prostate Cancer Cells

It has been discovered that by modulating the expression of genes linked to apoptosis in PC‐3 human prostate cancer cells, *J. regia* L. green husk extracts (chloroform, methanol, and n‐hexane) inhibited proliferation and promoted apoptosis in a dose‐ and time‐dependent manner. This included substantial alterations in the amounts of mRNA and the expression of related proteins, as well as DNA fragmentation. In PC‐3 cells treated with the extracts of walnut green husk, there was an apparent rise in the expression of the genes Bax, caspase‐3, and tumor protein p53 (TP53). Conversely, by exposure to the extracts, Bcl‐2 expression was suppressed (Alshatwi et al. 2012).

The walnut leaf hexane extract demonstrated potent and dose‐dependent anti‐proliferative effects on human prostate cancer cells. Significant apoptosis was also triggered by the extract in these PC3 cancer cells. After treatment with different concentrations of the extract, it significantly increased the development of apoptotic bodies. The population of cells in the sub‐G1 phase increased while the population of cells in the G2/M phase slightly decreased after 48 h of treatment with varying doses of the extract (Li et al. 2015). Besides, urolithin A, a walnut polyphenol metabolite, impact on lymph node carcinoma of the prostate (LNCaP) cells was investigated. It was shown that LNCaP cells had higher cyclin‐dependent kinase inhibitor 1A (CDKN1A) expression and lower 3‐phosphoinositide‐dependent kinase 1 (PDK1) expression, both of which have been connected to the development of cancer. LNCaP cells showed an increase in the population of apoptotic cells as well as cell cycle arrest (Sanchez et al. 2015).

##### 3.3.1.4 Breast Cancer Cells

Walnut leaf chloroform increased the cleavage of caspase‐3 and attenuated the proliferation as well as growth of Michigan Cancer Foundation‐7 (MCF‐7) cells (Salimi et al. 2014). Moreover, treating MCF‐7 cells with *J. regia* L. residual protein enhanced the rate of apoptosis and autophagy in these cells and decreased their growth (Ma et al. 2015). In addition, the effects of urolithin A on MCF‐7 breast cancer cells were investigated. Among the differentially expressed genes in MCF‐7 cells, decreased expression of PDK1 and up‐regulation of CDKN1A were observed. Up‐regulation of phosphatase and tensin homolog (PTEN) was also observed. MCF‐7 cells showed an increase in the number of apoptotic cells as well as cell cycle arrest (Sanchez et al. 2015).

Another in vitro study showed that a *J. regia* L. peptides‐zinc complex improved antiproliferative activity and decreased toxicity by combining the walnut peptides and zinc ions. Potent antiproliferative activity was demonstrated by the walnut peptides‐zinc complex against the chosen human cell lines, particularly MCF‐7 cells. The walnut peptides‐zinc complex caused cell apoptosis and cell cycle arrest, which decreased the growth of MCF‐7 cells. The findings showed that the ROS‐triggered mitochondrial‐mediated pathway and cell surface receptor‐mediated pathway were the mechanisms by which the walnut peptides‐zinc complex triggered apoptosis in MCF‐7 cells (Liao et al. 2016).

Furthermore, another investigation used three distinct solvents, methanol, ethanol, and hexane, to extract the constituents from walnut outer shells. The cytotoxic and antioxidant properties of the extracts were examined on MDA‐MB‐231 cells. It was discovered that walnut extracts significantly exhibited cytotoxic effects on the MDA‐MB‐231 cell line. It was also found that walnut outer bark had stronger anticancer properties than antioxidant activity (Dalkılıç et al. 2023).

##### 3.3.1.5 Colon Adenoma Cells

A research intended to determine whether different roasting conditions altered the chemo‐preventive advantages of *J. regia* L. Both raw and roasted walnuts went through in vitro digestion and fermentation. Following the application of fermentation supernatants to LT97 cells, the expression levels of CAT, glutathione S‐transferase pi (GSTP) 1, glutathione S‐transferase theta 2 (GSTT2), GSH‐Px1, and SOD2 genes, as well as cell proliferation and apoptosis, were investigated. Specifically, walnut fermentation supernatants boosted CAT and GSTT2 mRNA levels relative to the fermentation blank control, but GSH‐Px1 levels were significantly lowered. Adenoma cell proliferation was inhibited by walnut fermentation supernatants in a dose‐ and time‐dependent manner. Specifically, when compared to the blank control, larger concentrations of walnut fermentation supernatants substantially increased the amount of early apoptotic cells and triggered caspase‐3 activation. The effects that were found were not directly affected by the roasting procedure (Schlörmann et al. 2017).

##### 3.3.1.6 Keratinocytes

Researchers examined the effect of male *J. regia* L. flower on preventing UVB‐induced apoptosis in human skin cells (HaCaT cells). Before being exposed to UVB radiation, pretreatment with MEJR significantly decreases the risk of apoptotic characteristics, DNA damage, and decline in MMP (Muzaffer, Paul, Prasad, and Karthikeyan 2018).

##### 3.3.1.7 Colorectal Cancer Cell Line

A study was designed to explore the anti‐colorectal cancer effect and mechanism of green walnut husks. The potential ability of green walnut husks to prevent the proliferation, migration, and invasion of the mouse colon cancer cell line CT26, as well as trigger apoptosis, was discovered. Green *J. regia* L. husks were found to decrease tumor growth in the colorectal xenograft tumor model. Furthermore, it was discovered that the NOD‐like receptor family CARD domain containing 3 (NLRC3)/phosphoinositide 3‐kinase (PI3K)/AKT signaling pathway contributes to green walnut husks‐induced apoptosis in tumor cells (Chen et al. 2023).

#### 3.3.2 In Vivo

##### 3.3.2.1 Nervous System Disorders

The effects of walnut oil on hippocampal cell apoptosis and antioxidant activity in ovariectomized rats were studied. The ovariectomized rats had significantly lower serum estradiol (E2) levels than the young rats, whereas total SOD activity augmented in the walnut oil group with increasing doses. Meanwhile, in hippocampal dentate gyrus cells, wild walnut oil reduced MDA levels, increased Bcl‐2 expression, and decreased Bax expression. Furthermore, the dentate gyrus of hippocampal cells was protected from apoptosis (Chen et al. 2011) (Table 3).

Another investigation evaluated the potential of ethanolic extract of walnut to protect mice from cognitive impairment induced by a high‐fat diet. It was found that the ethanolic extract of walnut improved behavioral and memory impairment and decreased serum dyslipidemia, liver fat mass, and white adipose tissue. The antioxidant damage was prevented from the diabetic oxidative stress by the administration of ethanolic extract of walnut. The cholinergic system impairment was also suppressed. Additionally, by controlling the levels of MMP and formation of ROS in cerebral tissues, ethanolic extract of walnut repaired mitochondrial malfunction. Lastly, by cooperatively controlling the protein expression of the JNK signaling and apoptotic pathway (reducing Bax, caspase‐3, p‐JNK, increasing p‐AKT), ethanolic extract of walnut reduced brain damage (Moon, Kim, Lee, Kang, et al. 2022).

##### 3.3.2.2 Intestinal Injury

In mice, the radio‐protective potential of *J. regia* L. oligopeptides derived from walnut seed protein against damage caused by ^60^Coγ‐irradiation was studied. When compared to non‐administrated control mice, it was discovered that the administration of walnut oligopeptides enhanced the antioxidant defense system, facilitated hematopoietic recovery, and demonstrated a substantial capacity toward an increased survival rate and reduced weight loss. Walnut oligopeptides treatment also seems to be crucial in reducing intestinal epithelial barrier dysfunction, promoting epithelial integrity, and limiting ionizing radiation‐induced splenocyte apoptosis (decreasing the levels of Bax and caspase‐3, increasing the amounts of Bcl‐2) and inflammatory cascade (Zhu et al. 2019).

**FIGURE 3:**
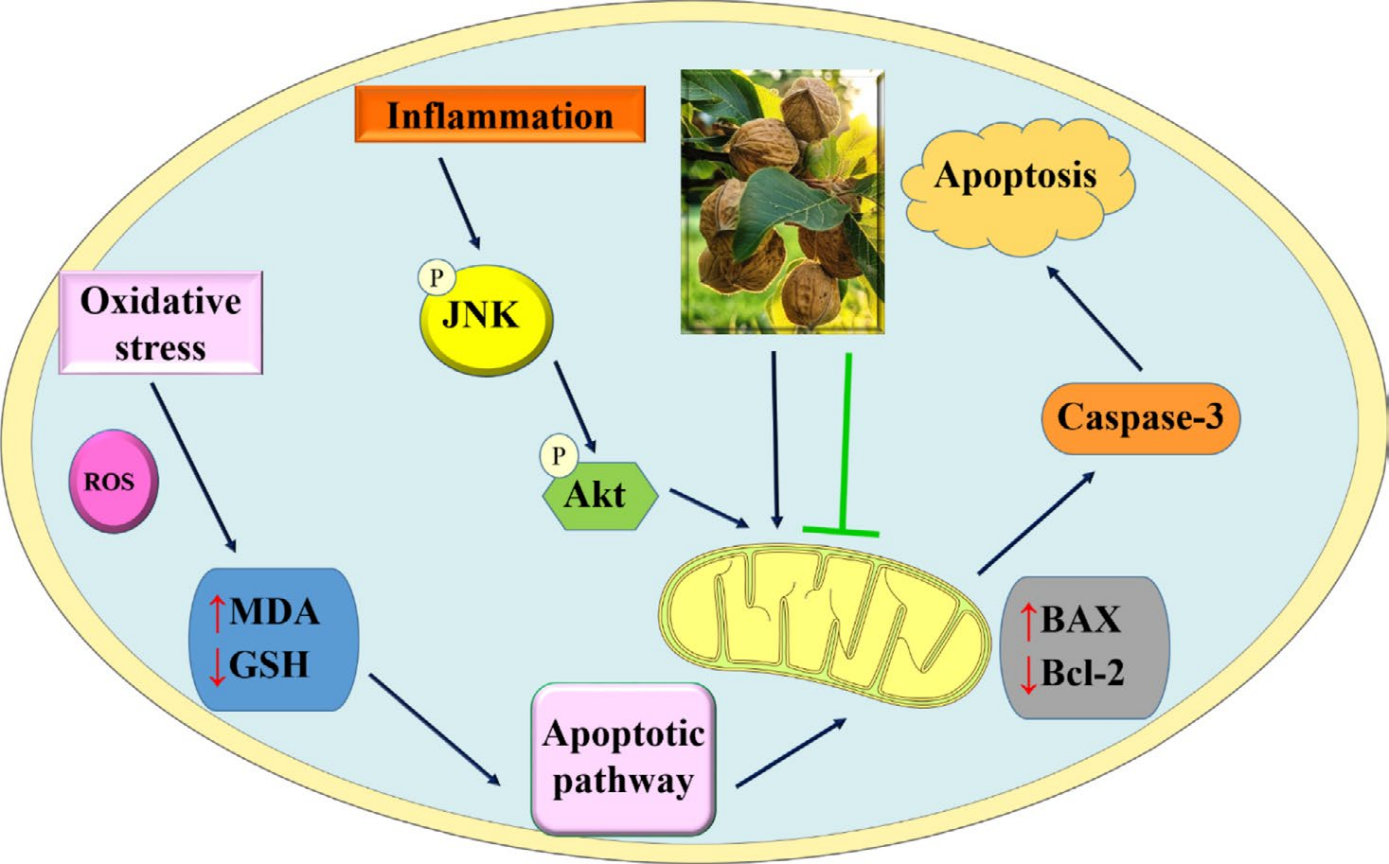
The apoptosis regulatory effects of *J. regia* L. (Images from https://smart.servier.com).

Taken together, the apoptosis regulatory effect of *J. regia* L. can be summarized as follows: numerous in vitro, in vivo, and clinical studies have been conducted to investigate the apoptosis regulatory properties of *J. regia* L., with a primary focus on various walnut green husk extracts, walnut peptides, as well as walnut kernel and leaf extracts. The in vitro studies presented a comprehensive overview of the anti‐proliferative and apoptosis‐inducing effects of various walnut extracts on different types of cancer cells and other cell models, including promyeloblasts, bone tumor cells, prostate cancer cells, breast cancer cells, colon adenoma cells, keratinocytes, and colorectal cancer cell lines. These findings underscore the potential of walnut derivatives in targeting cancer cells through mechanisms such as apoptosis induction, modulation of gene expression related to cell death pathways, and disruption of cell proliferation and migration. Walnut extracts showed modulation of key apoptotic genes like Bax, caspase‐3, and Bcl‐2 in the mentioned cells, leading to a dose‐dependent increase in apoptosis (Figure 3). The diverse range of effects observed in different cancer cell lines underscores the multifaceted nature of *J. regia* L. compounds in targeting cancer cells through multiple pathways. These studies lay a strong foundation for further exploration of walnut derivatives as potential anti‐cancer agents and highlight the importance of continued research to fully understand their mechanisms of action and therapeutic potential in cancer therapy.

The studies on walnut derivatives in animal models provide compelling evidence of their potential therapeutic benefits in addressing nervous system disorders and intestinal injuries. Walnut demonstrates its therapeutic properties by modulating antioxidant activity, apoptotic pathways, and promoting tissue recovery. These findings underscore the diverse pharmacological potential of walnut derivatives in mitigating cellular damage and promoting tissue resilience in complex biological systems.

However, further research encompassing in vivo validations, clinical trials, mechanistic explorations, and optimization of delivery methods is crucial to translate these findings into clinical practice effectively. Table 3 summarizes the properties of *J. regia* L. and its derivative on apoptosis.

### 3.4 Immunomodulatory Effects of Walnut and Its Main Components

The immune system protects the body from a variety of endogenous and external insults. Natural killer cells and phagocytes (macrophages, monocytes, neutrophils) are components of innate immunity, while memory cells are produced by the acquired immune system in response to an initial allergen, which enhances the body's reaction to repeated exposure to that particular allergen (Shakeri et al. 2024; Behrouz et al. 2022). Walnut has been shown to have immunomodulatory effects in a variety of immunological diseases.

#### 3.4.1 In Vitro

In mouse splenic cells, the effects of *J. regia* L. polyphenol extract on immunotoxicity triggered by 4‐pentylphenol and 3‐methyl‐4‐nitrophenol—two significant constituents of car emissions—were evaluated. Treatment with *J. regia* L. polyphenol extract was demonstrated to significantly increase the proliferation of splenocytes exposed to 4‐pentylphenol or 3‐methyl‐4‐nitrophenol, as evidenced by increases in the percentages of T cell subsets (cluster of differentiation 4 (CD4)^+^ and CD8^+^ T cells) and splenic T lymphocytes (CD3^+^ T cells), as well as the production of granzymes and T cell‐related cytokines (IL‐2, IL‐4, and granzyme‐B) in these cells. A reduction in oxidative stress was linked to these benefits, as shown by alterations in the levels of GSH‐Px, MDA, hydroxyl free radical (OH), and SOD (Yang et al. 2016) (Table 4).

**TABLE 4:** The immunoregulatory effects of *J. regia* L. on in vitro and in vivo experiments.

Compound	Study design	Doses/duration	Results	Ref.
In vitro				
Walnut polyphenol extract	Mouse splenic cells	0.01, 0.1, 1.0, 2.0, 3.0, 4.0, 5.0, 10.0 μg/mL	↑Splenocytes proliferation, CD4+, CD8+ T cells, CD3+ T cells, IL‐2, IL‐4, and granzyme‐B ↓Oxidative stress	(Yang et al. 2016)
In vivo				
Walnut septa aqueous extract	White mice	—	↑ Immature (band neutrophil), mature neutrophils counts in peripheral blood, division, differentiation, maturation of blast forms of myeloid and lymphoid line	(Dzidziguri et al. 2016)
Walnut vegetable oil	Male Wistar rats	0.9 g/kg, 10 days, gavage	↓TBARS, plasmatic lipase activity liver markers, lipid profiles	(Soussi et al. 2018)
Walnut oligopeptides	Female BALB/c mice	110, 220, 440 mg/kg, 30 days, p.o.	↑ T, Th cells, IL‐2, IL‐10, IL‐12, granulocyte–macrophage colony stimulating factor, IgA, IgG, IgM, and intestinal IgA, macrophage phagocytosis, natural killer cell activity	(Mao et al. 2020)
Walnut leaf ethanolic extract	Sprague Dawley rats	500 mg/kg, 12 days	↑ IL‐4 of blood ↓ Paw edema, arthritic development, blood levels of TNF‐α, NF‐κB, IL‐6, IL‐16, COX‐2, PGE2	(Mobashar et al. 2022)
Walnut leaf extract	*Oreochromis niloticus*	0, 250, 500, 750, 1000 mg/kg, 60 days, p.o.	↑ Serum SOD and CAT activity, myeloperoxidase and lysozyme activities, respiratory burst activity, potential activity, phagocytic index, phagocytic activity %, IL‐1β, IL‐8, IgM heavy chain gene expression, survival rate	(Yilmaz et al. 2023)

Abbreviations: CAT, catalase; CD, cluster of differentiation; COX‐2, cyclooxygenase‐2; Ig, immunoglobulin; IL, interleukin; NF‐κB, nuclear factor kappa B; PGE2, prostaglandin E2; Ref, references; SOD, superoxide dismutase; TBARS, thiobarbituric acid reactive substances; TNF‐α, tumor necrosis factor alpha.

#### 3.4.2 In Vivo

The immune‐regulatory effects of *J. regia* L. septa aqueous extract were evaluated using a mouse leukopenia experimental model. It has been demonstrated that the walnut septa extract can restore inhibited myelopoiesis, which was induced by cyclophosphamide injection. The process of normalizing blood formula through the use of the extract was facilitated by the rapidly rising count of immature (band) and mature neutrophils in peripheral blood. In the bone marrow of mice with leukopenia, it has been demonstrated that *J. regia* L. septa extract increases the division, differentiation, and maturation of blast forms of myeloid as well as lymphoid line (Dzidziguri et al. 2016).

An investigation into the effectiveness of walnut vegetable oil against lead‐induced hepatotoxicity was conducted. Lead administration raised the levels of lipid profiles TC, TG, VLDL‐C, LDL‐C levels, thiobarbituric acid reactive substances (TBARS), nitrogen oxides, and protein carbonyl, plasmatic lipase activity, and inflammatory markers, but decreased plasmatic ALP. Hepatic markers (LDH, ALT, AST, and bilirubin) and lipid profiles were also elevated. Co‐administration of walnut *J. regia* L. oil decreased the rise of TBARS and plasmatic lipase activity while restoring all liver markers, lipid profiles, and antioxidants to near‐normal levels. Studies on hepatic histology verified the positive effects of walnut vegetable oil by improving every biochemical parameter (Soussi et al. 2018).

An in vivo investigation explored the immunomodulatory effects of walnut oligopeptides in mice. The results showed that *J. regia* L. oligopeptides could considerably increase cell‐mediated and humoral immune responses, natural killer cell activity and macrophage phagocytosis. Furthermore, elevated cytokines secretion of IL‐2, IL‐10, IL‐12, T and Th cells percentages, and granulocyte–macrophage colony stimulating factor, intestinally released IgA, and synthesis of IgA, IgG, IgM played a part to these advantages (Mao et al. 2020).

Another research examined the anti‐arthritic properties of an ethanolic leaf extract from walnuts in rodents. The extracts not only reduced arthritic progression and reduced paw swelling, but they also improved a variety of tissue examination outcomes. Pro‐inflammatory cytokines and COX‐2 were reduced, but IL‐4 was raised. Additionally, PGE2 levels declined in the groups treated with the extract. The physiological and biochemical signs in the therapy groups had practically returned to the normal range. Furthermore, the extract significantly reduced paw edema caused by dextran, serotonin, histamine, and carrageenan (Mobashar et al. 2022).

Administration of *J. regia* L. leaf extract affects *Oreochromis niloticus* development, immunity, and resistance to bacterial infection. After feeding the fish with walnut diets for 60 days, *Plesiomonas shigelloides* was introduced. Prior to the challenge, liver function enzymes (AST and ALT) activities, blood proteins (total protein, albumin, globulin), and growth were found to be unaffected by dietary walnut leaf extract. Compared to other groups, the walnut leaf extract 250 mg/kg group showed a significantly greater rise in serum SOD and CAT activity. When comparing the walnut leaf extract groups to the control group, there was a substantial rise in the serum immunological indices (myeloperoxidase and lysozyme activities) and hematological parameters (respiratory burst activity, potential activity, phagocytic index, phagocytic activity %). Compared to the control group, all walnut leaf extract‐supplemented groups had significantly higher levels of IL‐1β, IL‐8, and IgM heavy chain gene expression, as well as a higher survival rate (Yilmaz et al. 2023).

#### 3.4.3 Clinical Trials

In allergic and non‐allergic individuals, the effects of walnut focused on reactive T‐cells and their phenotypic characteristics and frequencies in relation to the disease. Jug r 2 was identified as the main allergen that elicits CD4 (^+^) T‐cell responses. Multiple T‐cell epitopes were found inside Jug r 2. Most allergic participants' responsive T cells had a C‐C chemokine receptor type 4 (CCR4) (^+^) phenotype, with a subset expressing CCR4(^+^) CCR6(^+^), regardless of asthma status. Cytokine staining indicated a variety of profiles, including Th2‐, Th2/Th17‐, and Th17‐like responses. T‐cell clones from allergic patients primarily expressed GATA‐binding protein 3, with some additionally expressing RAR‐related orphan receptor C (RORC), indicating the presence of Th2, Th2/Th17, and Th17 cell subsets (Archila et al. 2015).

**FIGURE 4:**
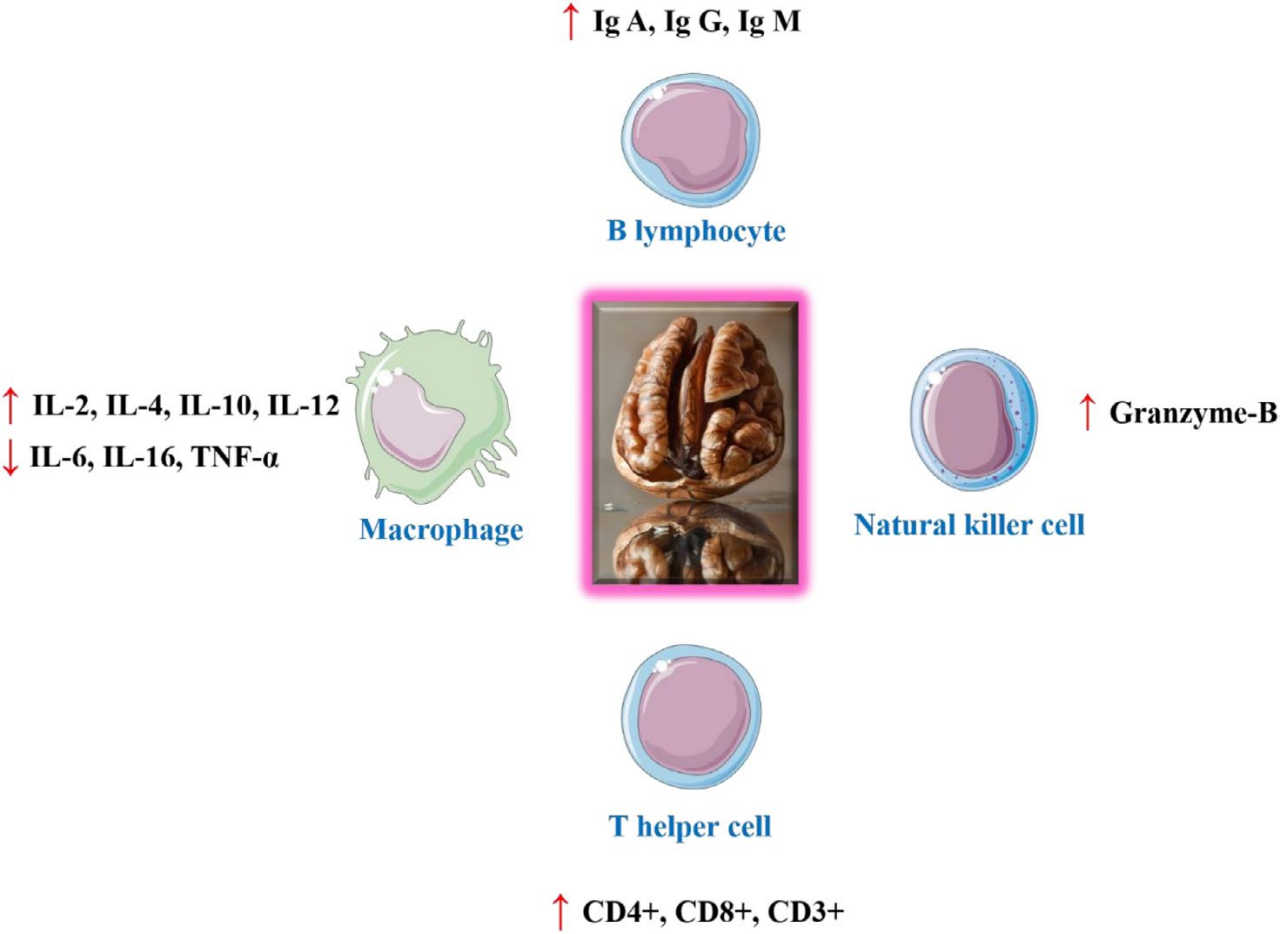
The immunomodulatory effects of *J. regia* L. (Images from https://smart.servier.com).

Taken together, the immunomodulatory impact of *J. regia* L. can be summed up as follows: a number of in vitro, in vivo, and clinical investigations have been carried out to explore the immunomodulatory qualities of *J. regia* L., with a particular emphasis on extracts from walnut leaves. In the provided text, various studies have investigated the immunomodulatory effects of walnut extracts, their potential therapeutic benefits, and their impact on health parameters. These studies have highlighted the positive immunomodulatory properties of walnut derivatives, showing their ability to enhance immune responses, reduce oxidative stress, and alleviate inflammatory cons (Figure 4). However, it is important to consider some limitations in these findings. Many of the studies are based on animal models, which may not directly correlate with human responses. Additionally, the specificity of focusing on walnut‐derived compounds limits the generalizability of the results to other interventions. Furthermore, the short‐term nature of the studies raises questions about the long‐term effects and potential side effects of sustained walnut extract consumption.

For future research directions, it is suggested that further clinical trials be conducted to validate the immunomodulatory effects of walnut extracts in human subjects. Mechanistic studies can provide a deeper understanding of the molecular pathways through which walnut extracts exert their effects, guiding targeted therapeutic approaches. Comprehensive safety assessments are also necessary to evaluate the long‐term implications of walnut extract consumption.

It is important to note that walnuts themselves can be allergenic; thus, caution should be exercised in individuals with known nut allergies. In short, while the studies demonstrate the promising immunomodulatory and therapeutic potential of *J. regia* L.‐derived compounds, additional research in clinical settings is essential to confirm their efficacy, safety profile, and broader applicability in enhancing immune responses and managing inflammatory conditions. Table 4 summarizes immune‐regulatory properties of *J. regia* L. and its derivative.

## 4 Conclusion

In the present study, the aim was to investigate the multifaceted therapeutic properties of *J. regia* L., shedding light on their antioxidant, anti‐inflammatory, apoptosis‐regulatory, and immunomodulatory effects. Walnut (mainly walnut kernel and walnut kernel extracts) exhibits potent antioxidant properties attributed to its ability to scavenge ROS, prevent DNA damage, and maintain cellular redox balance. These effects are crucial in protecting various cell types from oxidative stress–induced damage. Mechanistically, *J. regia* L. modulates antioxidant enzyme activity, reduces ROS levels, inhibits protein oxidation and lipid peroxidation, and helps attenuate oxidative stress–related harm. The diverse array of antioxidant effects observed in studies suggests that *J. regia* L. could be beneficial in managing a spectrum of ailments, including nervous system disorders, lung injuries, diabetes, hepatotoxicity, and arthritis.

Walnut‐derived components (mostly walnut kernel, walnut kernel extracts, and walnut peptides) have also demonstrated significant anti‐inflammatory effects by targeting key inflammatory mediators, signaling pathways, and cellular processes across various systems. These components modulate inflammation and oxidative stress markers, potentially improving conditions like arthritis, obesity, and diabetes. Mechanistic insights reveal that *J. regia* L. compounds can enhance autophagy, cognitive function, and reduce inflammation by modulating gut microbiota and metabolic profiles. The multifaceted nature of walnut's anti‐inflammatory effects suggests its potential in managing chronic inflammatory conditions through diverse pathways.

Moreover, *J. regia* L. extracts exhibit promising apoptosis‐inducing effects on various cancer cell lines, implicating their potential as anti‐cancer agents. These extracts modulate apoptotic pathways by influencing key genes like Bax, caspase‐3, and Bcl‐2, leading to dose‐dependent increases in apoptosis. Walnut compounds disrupt cell proliferation and migration, while also influencing gene expression related to cell death pathways. The broad spectrum of effects observed indicates the multifaceted nature of walnut compounds in targeting cancer cells through diverse mechanisms, highlighting their potential in cancer therapy. Notably, walnut (chiefly the green husk extracts, walnut peptides, and walnut kernel extracts) demonstrates a dual role in apoptosis regulation, selectively inducing apoptosis in cancer cells while protecting healthy cells from apoptosis, showcasing its tailored response based on the cellular context.

In addition, *J. regia* L. derivatives (largely walnut leaf extracts) possess positive immunomodulatory properties that enhance immune responses, reduce oxidative stress, and alleviate inflammatory conditions. These effects could be beneficial in managing various health parameters. Studies suggest that walnut extracts can positively impact inflammation and immune responses, potentially offering therapeutic benefits in immune‐related conditions. However, it is essential to note that despite these benefits, walnuts can trigger allergic reactions in susceptible individuals.

While these studies collectively demonstrate the antioxidant, anti‐inflammatory, apoptosis‐regulatory, and immunomodulatory properties of walnuts, it is essential to address limitations such as the need for further research in human populations, identification of bioactive compounds, standardized dosages, and long‐term safety studies. By incorporating these suggestions into future research, a more comprehensive understanding of the therapeutic potential of *J. regia* L. as a natural therapeutic agent can be achieved, paving the way for innovative preventive and therapeutic interventions in various health conditions.

## Author Contributions

**Mahboobeh Ghasemzadeh Rahbardar:** investigation (equal), writing – original draft (lead). **Mostafa Rashki:** writing – review and editing (supporting). **Mohammad Hossein Boskabady:** conceptualization (lead), project administration (lead), writing – review and editing (lead).

## Ethics Statement

The authors have nothing to report.

## Consent

The authors have nothing to report.

## Conflicts of Interest

The authors declare no conflicts of interest.

## Data Availability

No new data were created or analyzed during this study. Data sharing is not applicable to this article.
